# Cognitive-Motor Duality in Human M1 and Cerebellum

**DOI:** 10.21203/rs.3.rs-10164419/v1

**Published:** 2026-09-10

**Authors:** Chen Zheng, Shitong Xiang, Chao Xie, Xuefei Wang, Runye Shi, Yechen Hu, Zhengyu Yang, Qing Lin, Tobias Banaschewski, Gareth J. Barker, Arun L.W. Bokde, Rüdiger Brühl, Sylvane Desrivières, Herta Flor, Penny Gowland, Antoine Grigis, Andreas Heinz, Herve Lemaitre, Jean-Luc Martinot, Marie-Laure Paillère Martinot, Eric Artiges, Frauke Nees, Dimitri Papadopoulos Orfanos, Luise Poustka, Michael N. Smolka, Sarah Hohmann, Nilakshi Vaidya, Henrik Walter, Robert Whelan, Paul Wirsching, Jie Zhang, Xing-Ming Zhao, Jianfeng Feng, Gunter Schumann, Tianye Jia, ZIB Consortium and IMAGEN Consortium

**Affiliations:** 1. Institute of Science and Technology for Brain-Inspired Intelligence (ISTBI), Fudan University, Shanghai, China; 2. Key Laboratory of Computational Neuroscience and Brain-Inspired Intelligence, Fudan University, Ministry of Education, Shanghai, China; 3. Department of Psychological and Cognitive Sciences, Tsinghua University, Beijing, China; 4. School of Data Science, Fudan University, Shanghai, China; 5. Department of Child and Adolescent Psychiatry and Psychotherapy, Central Institute of Mental Health, Medical Faculty Mannheim, Heidelberg University, Square J5, 68159 Mannheim, Germany; German Center for Mental Health (DZPG), partner site Mannheim-Heidelberg-Ulm; 6. Department of Neuroimaging, Institute of Psychiatry, Psychology & Neuroscience, King’s College London, United Kingdom; 7. Discipline of Psychiatry, School of Medicine and Trinity College Institute of Neuroscience, Trinity College Dublin, Dublin, Ireland; 8. Physikalisch-Technische Bundesanstalt (PTB), Braunschweig and Berlin, Germany; 9. Social, Genetic and Developmental Psychiatry Centre, Institute of Psychiatry, Psychology & Neuroscience, King’s College London, United Kingdom; 10. Institute of Cognitive and Clinical Neuroscience, Central Institute of Mental Health, Medical Faculty Mannheim, Heidelberg University, Square J5, Mannheim, Germany; 11. Department of Psychology, School of Social Sciences, University of Mannheim, 68131 Mannheim, Germany; 12. Sir Peter Mansfield Imaging Centre School of Physics and Astronomy, University of Nottingham, University Park, Nottingham, United Kingdom; 13. NeuroSpin, CEA, Université Paris-Saclay, F-91191 Gif-sur-Yvette, France; 14. Department of Psychiatry and Psychotherapy, University of Tübingen, Germany; German Center for Mental Health (DZPG), Site Tübingen, Germany; 15. Department of Psychiatry and Psychotherapy, Technische Universität Dresden, Dresden, Germany; 16. Institut des Maladies Neurodégénératives, UMR 5293, CNRS, CEA, Université de Bordeaux, 33076 Bordeaux, France; 17. INSERM U1299 “Developmental Trajectories in Psychiatry & Addictology”; University Paris-Saclay, Ecole Normale Supérieure Paris-Saclay, Mathematics department, Centre Borelli CNRS UMR9010; Gif-sur-Yvette; France; 18. Department of Psychiatry, LabD-Psy, Barthélémy Durand Hospital, Etampes, France; 19. AP-HP. Sorbonne Université, Department of Child and Adolescent Psychiatry, Pitié-Salpêtrière Hospital, Paris, France; 20. Institute of Medical Psychology, Ludwig-Maximilians-Universität (LMU) in Munich, Munich, Germany; 21. Department of Child and Adolescent Psychiatry, Center for Psychosocial Medicine, University Hospital Heidelberg, Heidelberg, Germany; 22. Centre for Population Neuroscience and Stratified Medicine (PONS), Department of Psychiatry and Psychotherapy, Charité Universitätsmedizin Berlin, Germany; 23. Department of Psychiatry and Psychotherapy CCM, Charité – Universitätsmedizin Berlin, corporate member of Freie Universität Berlin, Humboldt-Universität zu Berlin, and Berlin Institute of Health, Berlin, Germany; 24. School of Psychology and Global Brain Health Institute, Trinity College Dublin, Ireland; 25. Department of Neurology, Zhongshan Hospital, Fudan University, Shanghai, China; 26. College of Biomedical Engineering, Fudan University, Shanghai, China; 27. State Key Laboratory of Brain Function and Disorders, Fudan University, Shanghai, China; 28. MOE Frontiers Center for Brain Science, Fudan University, Shanghai, China; 29. Department of Cardiovascular Medicine, RuiJin Hospital Lu Wan Branch, Shanghai Jiaotong University School of Medicine; 30. Huzhou Central Hospital, Affiliated Central Hospital Huzhou University, Huzhou, Zhejiang 313000, China; 31. School of Mathematical Sciences and Centre for Computational Systems Biology, Fudan University, Shanghai, China; 32. Department of Computer Science, University of Warwick, Coventry, UK; 33. Centre for Population Neuroscience and Stratified Medicine (PONS), Institute for Science and Technology of Brain-Inspired Intelligence and National Centre for Neurological Disorders, Huashan Hospital, Fudan University, Shanghai, China; 34. German Center for Mental Health (DZPG), Site Berlin-Potsdam, Germany; 35. National Institute on Drug Dependence, Peking University, Beijing, China; 36. Beijing Key Laboratory of Innovative Intervention Techniques and Translational Research for Mental Disorders Rehabilitation, Peking University, Beijing, China; 37. Psychology Department, University of Southampton, Southampton, UK

## Abstract

The translation of abstract cognitive intention into localised physical action is a defining feature of voluntary behaviour^1,2^. To achieve this, the brain must maintain a highly regulated preparatory state, yet the macroscopic architecture of this interface remains elusive. Historically, the primary motor cortex has been viewed simply as a strictly contralateral kinematic relay^3^, making its robust bilateral activation during single-limb preparation a long-standing functional paradox^4,5^. Here, we show that human motor preparation is driven by two distinct, superimposed neural processes operating across the cerebral cortex and cerebellum. By mathematically decomposing neuroimaging signals from over six thousand sessions, we identified a broadly distributed cognitive readiness network that dictates the precise timing of an action, and an independent, highly localised kinematic preparation network that controls execution speed. This macroscopic duality maps human whole-brain dynamics onto the neural subspaces and circuits previously observed only at the microscopic level in animal models^6-9^. Furthermore, we reveal that genetic risk for Parkinson’s disease selectively impairs this cognitive readiness network during healthy adolescence. Ultimately, these findings redefine the cortico-cerebellar axis as an active computational interface integrating abstract thought with physical embodiment, offering a generalisable framework for understanding mind-body synchronisation and early neurodegenerative vulnerability.

## Introduction

The translation of an abstract cognitive intention (when to act) into a highly localised physical command (how to act) is a defining feature of voluntary behaviour. To achieve this, the central nervous system must maintain a state of high-alert cognitive readiness without leaking signals that prematurely trigger muscle execution. In non-human primates, this mind-body interface has been described mathematically at the population level of neurons: cortical preparatory dynamics evolve in a movement-invariant, 'output-null' subspace before transitioning into an execution-driving 'output-potent' subspace^6,7^. However, how this abstract cognitive state is anatomically superimposed onto the physical execution machinery in the human brain, and how it is broadcast across large-scale cortical and subcortical networks, remains a central puzzle in understanding the human mind-body interface.

For decades, the primary motor cortex (M1) has been conceptualised as the rigid anatomical boundary between cognition and physical execution. Historically modelled as a downstream puppet, human M1 is classically depicted as a highly localised, strictly contralateral relay adhering to Penfield’s homuncular map^3^. This traditional view is currently undergoing a paradigm shift. Recent high-resolution neuroanatomical mapping reveals that M1 is not a monolithic kinematic structure; instead, classic effector-specific regions are physically interleaved with a higher-order, effector-nonspecific Somato-Cognitive Action Network (SCAN) linked to generalised action planning^10^. While this alternating map highlights M1's broader cognitive role, a fundamental mechanistic gap remains: how do these anatomically interleaved networks functionally communicate to execute a simple movement? The transition from intention to execution cannot occur if abstract intention and physical output are completely isolated from one another. To execute a precisely timed movement, the global cognitive state (when to act) must directly synchronise with the execution machinery (how to act). This suggests that classical effector zones are unlikely to be blind, purely kinematic islands, which is supported by recent single-neuron recordings in humans showing that complex, whole-body movement representations extend well beyond the SCAN boundaries, residing directly within classical effector regions^11^. Thus, they must act as integrative hubs where brain-wide cognitive readiness dynamically superimposes upon local physical commands^1,2,11^. Crucially, this precise spatiotemporal synchronisation cannot be achieved by the cortex alone; it intrinsically necessitates the cerebellum to act as a coincidence detector for motor initiation, bridging the widespread intention network with localised kinematic loops^12,13^.

The clearest and most enduring empirical clue pointing towards this dual role is the phenomenon of bilateral M1 activation during strictly unimanual tasks^4,5,14^. Under the classical contralateral execution model, this robust ipsilateral engagement is a physiological paradox. Previous literature has heavily debated its function, with some models attributing it to interhemispheric inhibition necessary to suppress mirror movements, and others arguing for active facilitation, suggesting the ipsilateral cortex continuously encodes kinematic scaling^15-17^. At the population level of neurons, recordings indicate that while signals for each arm are heavily mixed across hemispheres, they ultimately segregate into distinct subspaces to allow downstream kinematic decoding^18,19^. Ultimately, these competing models fail to capture the broader cognitive imperative. Bilateral M1 engagement is likely not a simple mechanical byproduct of either mirror-movement suppression or assistive facilitation alone. Rather, it may reflect a simultaneous integration of both: the global broadcast of cognitive intention dynamically superimposed upon the strictly lateralised, competitive demands of physical execution.

Here, we bridge these frameworks by leveraging the largest longitudinal and cross-sectional fMRI cohorts to date (IMAGEN and ZIB, n>6000 sessions)^20,21^. We posit that unilateral motor preparation spatiotemporally integrates two distinct neural systems across a unified cortico-cerebellar axis. By decomposing bilateral M1 and cerebellar preparatory signals, we identified two orthogonal latent components: a hand-invariant cognitive readiness signal and a hand-specific kinematic preparation signal^22,23^. Crucially, these decomposed signals reveal a striking behavioural double-dissociation: the cognitive readiness signal reflects a bi-hemispheric coalition, recruiting a broad cortico-striato-thalamic network matching the Somato-Cognitive Action Network (SCAN)^10^, to govern the temporal precision of when to act; conversely, the kinematic preparation signal, omitting SCAN, represents a highly lateralised command that specifically controls reaction kinematics (how to act) while suppressing the ipsilateral hemisphere. By bridging the macroscopic SCAN architecture with principles derived from microscopic population dynamics, this cognitive-motor dual system reframes M1 and the cerebellum not merely as motor relays, but as active computational hubs essential for the optimal integration of thought and action.

## Results

### Bilateral activations in M1 during the motor preparation

We investigated unimanual motor preparation in the longitudinal IMAGEN cohort. Participants performed a Monetary Incentive Delay (MID) task requiring left- or right-hand responses at ages 14 (BL, N=1601), 19 (FU2, N=1143), and 23 (FU3, N=953) (Fig. 1a). During anticipation, robust activation consistently occurred in both contralateral and ipsilateral M1 across timepoints and reward conditions (Fig. 1c, and Table S1).

To evaluate this bilateral engagement, we defined functional regions of interest (ROIs) within the M1 hand-knob by intersecting significantly activated voxels across timepoints and conditions (Fig. 1d). Contralateral signals were expectedly stronger than their ipsilateral counterparts (*P*<4.8×10^−105^, Table S2). Strikingly, contralateral and ipsilateral M1 activations were strongly positively correlated across conditions and hands (Fig. 1, e to g, and Fig. S1). However, this tight interhemispheric coupling occurs despite the hemispheres exhibiting opposing relationships to subsequent motor execution, presenting a functional paradox.

### Opposing behavioural associations reveal a hidden functional duality in M1

Linear mixed-effects models (LMMs) evaluated how preparatory M1 signals predict reaction time (RT) in successful trials. Independently, contralateral M1 activation predicted faster responses (e.g., right-hand β_std_=−0.06, *P*=1.4×10^−10^), whereas ipsilateral M1 predicted slower responses (β_std_=0.06, *P*=1.9×10^−11^) (Fig. S2 and Table S3). Modelled simultaneously, mutual statistical suppression strengthened their opposing behavioural associations (contralateral Δβ_std_=−0.025; ipsilateral Δβ_std_=0.024; both empirical *P*_bootstrap_ < 0.0001) (Table S3). Consequently, incorporating ipsilateral activity improved RT prediction over contralateral signals alone (Table S4).

This mutual enhancement indicates that bilateral M1 activations share substantial common variance orthogonal to their lateralised contributions to execution speed. Remarkably, this functionally divergent dynamic remained highly stable across timepoints and conditions, and successfully generalised across hands (e.g., BL right-hand contralateral activation predicted left-hand RT; r=−0.16, *P*=5.9×10^−10^) (Fig. 1h and Fig. S3).

Thus, a physiological paradox emerges: bilateral M1 regions co-activate during preparation yet competitively influence kinematic output, establishing that single-limb motor preparation is not a monolithic event. Instead, it superimposes two distinct mechanisms: a shared state (underlying the co-activation) and a lateralised competitive gradient (driving the execution speed). To resolve this, we computationally decomposed the bilateral M1 signals into two latent components.

### Latent signal decomposition reveals a cognitive-kinematic dichotomy in M1

Using multivariate linear regression (MLR), we decomposed bilateral M1 signals into two orthogonal latent components: a hand-invariant shared component capturing common bi-hemispheric variance, and a hand-specific discriminative component reflecting the interhemispheric activation gradient (Fig. 2a). This decomposition proved highly robust across all timepoints, exhibiting strong intra-class correlations (0.51<r<0.64, *P*<1.1×10^−54^) and weak to negligible inter-class correlations (∣r∣<0.12, *P*>3.8×10^−4^) (Fig. 2b and Fig. S4, a to c).

Over 10 years, the discriminative signal demonstrated higher longitudinal reliability than the undecomposed contralateral signal (e.g., BL vs. FU2: mean r_contralateral_=0.11, mean r_discriminative_=0.27, 3.46<Z, *P*<2.7×10^−4^, Dunn and Clark's Z-test, Table S5), isolating a stable, trait-like functional architecture resilient to state noise. Conversely, the shared component mirrored the temporal stability of the undecomposed BOLD signal (e.g., BL vs. FU2: mean r_contralateral_=0.11, mean r_shared_=0.13, ∣Z∣<1.36, *P* > 8.7×10^−2^, Table S5), reflecting state-dependent fluctuations in sustained attention.

Crucially, these latent components exhibited a striking double dissociation with task performance (Fig. 2, c to h, and Fig. S4, d and e). The discriminative signal associated exclusively with execution speed, consistently predicting faster RT only during successful trials across timepoints (e.g., BL: r<−0.15, *P*=1.0×10^−8^), thus in line with classical kinematic motor preparation. In contrast, the shared signal dictated global response precision, with higher shared component robustly predicting a more concentrated, temporally precise response (e.g., for Big-win at BL: standard deviation of overall RT, r=−0.14, *P*=2.4×10^−8^, Fig. 2, f to i, and Fig. S4, f and g). Because minimising overall reaction time variability and preventing premature errors requires sustained vigilance and probabilistic timing, extending well beyond traditional motor readiness mechanisms like postural adaptation and sensory gating, this shared signal in M1 represents a higher-order cognitive computation of when to act.

Together, these findings establish a cognitive-motor dual regulatory system within human M1. The bi-hemispheric shared component governs cognitive readiness, determining when to act by tuning global temporal precision and vigilance. Conversely, the lateralised discriminative component acts as kinematic preparation, dictating how fast to act once the temporal gate is opened. Macroscopically, cognitive readiness dominates overall task performance, whereas kinematic preparation only optimises speed in successful trials. Microscopically, this macroscopic duality mirrors the division of labour in layer 5 projection neurons. The shared signal aligns with dominant movement-invariant PT population dynamics, likely initiated by the inter-hemispheric movement-invariant IT neurons to establish movement intention. Conversely, the discriminative signal mirrors movement-specific preparation routed through specialised IT sub-populations, which ultimately descends via lateralised, medullary-projecting PT_lower_ neurons for execution^8,9,12^.

### Converging evidence from model-free signal decomposition

To confirm these dual processes reflect intrinsic neural axes rather than model artefacts, we applied model-free principal component analysis (PCA) to the four M1 hand-region signals (hemispheres × responding hands) across participants. The top two principal components (PCs) replicated our model-derived latent components (Table S6). PC1 captured a generalised, hand- and hemisphere-invariant signal with approximately equal loadings (~50% explained variance), correlating almost perfectly with the model-derived shared signal (e.g., BL Big-win: r > 0.99). PC2 isolated the precise contralateral-versus-ipsilateral contrast (~20% explained variance), directly mirroring the model-based discriminative signal (e.g., BL Big-win: r > 0.99). The remaining minor PCs (capturing isolated hemispheric or hand-specific signals) exhibited no consistent behavioural associations (Figs. S5 and S6).

We next investigated whether this dual-system architecture is a general feature of the broader motor network by extending the decomposition to the ventral and dorsal premotor cortices (PMv, PMd), supplementary motor area (SMA), preSMA, and the full M1 ^24^. While the shared PC1 consistently explained half of the variance across all extended regions, the discriminative PC2 degraded precipitously outside the M1 hand area, dropping to <8.1% explained variance with highly unstable cross-condition reliability (Fig. S7).

This topological divergence provides critical mechanistic insight into the hierarchical organisation of motor preparation. While the shared component persists across the preSMA, SMA, and premotor cortices, indicating that cognitive readiness is a globally broadcast state, M1’s predictive contribution remains robustly independent when all regions are evaluated simultaneously (e.g., for Big-win deviation of overall RT: β_std_<−0.14, *P*<4.1×10^−3^ across timepoints; tables S7 and S8). This resilient effect subverts the classical top-down model: rather than serving merely as a passive downstream kinematic relay, M1 also operates as an independent cognitive hub to actively govern optimal action timing. In contrast, kinematic preparation is a highly localised, effector-specific computation strictly anchored to the M1 hand area (tables S7 and S8).

### Brain-wide involvement of both latent regulatory processes

To map the brain-wide architecture of these dual regulatory processes, we assessed each latent component’s contribution to global voxel activations. The shared component exhibited broad, positive, and symmetrical recruitment across classic motor-related networks, including the bilateral motor cingulate, putamen, and cerebellar hand areas (Fig. 3). Notably, this global cognitive readiness network included the putaminal fundus (Fig. 3i), a core ventral subcortical hub critically implicated in the early neurodegenerative symptoms of Parkinson’s disease^25,26^. The discriminative component engaged a spatially overlapping network but exhibited a strict cross-hemispheric push-pull organisation, characterised by positive contralateral and negative ipsilateral associations (Fig. 3). Despite their spatial overlap, the shared network extended far beyond discriminative boundaries. Strikingly, their brain-wide spatial divergence was predominantly driven by the shared cognitive readiness network and mapped precisely onto the recently discovered Somato-Cognitive Action Network (SCAN), which also originates within M1 (Fig. 3)^10^. Rather than discrete cognitive-only intervals, our results realign SCAN into a broader M1-spanning cognitive readiness network, serving as primary cortical nodes that structurally and functionally separate global cognitive intention from lateralised kinematic execution, where the hand-area M1 ultimately serves as the primary gateway for kinematic preparation.

### Decomposition using single-hand M1 activations

To verify the dual system in hand-specific trials, we repeated the decomposition restricted to either left- or right-hand trials. The dissociation between shared and discriminative components remained robust, exhibiting strong intra-class (discriminative: r>0.51, *P*<1.0×10^−56^, shared: r>0.36, *P*<3.6×10^−28^) and negligible inter-class correlations (mean ∣r∣=0.06) (Fig. S8). Behavioural associations were similarly preserved (Figs. S9 and S10). Crucially, cross-hand comparisons revealed that while shared signals were highly correlated between hands (r>0.36, *P*<2.2×10^−31^), discriminative signals were structurally independent (−0.07<r<0.13) (Fig. S8). This strongly supports a global, hand-invariant cognitive readiness mechanism versus segregated hand-specific execution commands.

Unexpectedly, these largely independent hand-specific discriminative signals still predicted the RT of successful trials for the opposite hand (−0.21<r<−0.08, 4.3×10^−10^<*P*<1.1×10^−2^) (Figs. S9 and S10). This paradox reveals a critical distinction between computational representation and biological substrate. The mathematical orthogonality between the left and right discriminative signals acts as a forced computational mechanism to suppress the unused limb. However, their cross-predictive power suggests they originate from a highly intermixed neural population rather than strictly segregated pools. This aligns with recent human microelectrode recordings, which demonstrate that representations of different limbs are intrinsically interlinked within localised effector zones^11^. This macroscopic dynamic parallels the micro-functional organisation of layer 5 projection neurons, where dominant movement-invariant population dynamics establish readiness, while only a sparse, distributed subnetwork routes movement-specific kinematic commands^9^.

### Mirrored dual-regulation model in the cerebellum

Given tight M1-cerebellum coupling^27-29^, we investigated whether this dual-system architecture extends to the cerebellar hand-motor areas. Applying identical signal decomposition to cerebellar ROIs, defined by robust discriminative activations (Fig. 4a), we replicated the M1 functional dissociation. Distinctly, the cerebellar discriminative latent signals selectively predicted RT during successful trials (e.g., BL: 0.10<r<0.19, 2.5×10^−13^<*P*<1.3×10^−4^) (Fig. 4, b to f, and Fig. S11). Conversely, the bi-hemispheric shared latent signals broadly tracked global temporal precision, significantly correlating with overall reaction time, its standard deviation, success rate, and both premature and delayed error rates (e.g., BL right-hand response for Big-win: standard deviation of overall RT: r=−0.15, *P*=6.7×10^−9^) (Fig. 4, c and g to i, and Figs. S11 and S12). These findings confirm that the cognitive readiness (when) and kinematic preparation (how) dichotomy is not a local cortical phenomenon, but a system-wide property of the cortico-cerebellar network. Crucially, this mirrored functional segregation supports the cerebellum's essential mechanistic role in motor initiation: acting as a functional hub to synchronise the bilateral cognitive intent with the lateralised physical bias^12,13^.

### Generalisability of the dual-regulation model in the ZIB cohort

To assess the generalisability and dynamic predictive capacity of our dual-regulation model, we extended our analysis to the Zhangjiang International Brain BioBank (ZIB) cohort (N=2,638 participants). Unlike IMAGEN, the ZIB task featured highly variable delay durations (1.5–4.0s). To dissect how temporal expectancy modulates preparation, we categorised trials by delay difference (Δ) relative to the preceding trial: compressed (Δ<0s), consistent (0≤Δ≤1s), and extended (Δ>1s) (Fig. 5a)^30,31^.

Behaviourally, temporal prediction errors yielded asymmetric impairments. Compressed trials induced universal unpreparedness (too-late errors) (Fig. 5, b and d, and Table S9). Extended trials, however, caused a generalised loss of precision, polarising the cohort into hyper-anticipatory (too-early) and inattentive (too-late) responders (Fig. 5, c and d, and Table S9).

Applying PCA, we robustly reproduced the two latent M1 components across all temporal contexts. The shared cognitive readiness signals consistently predicted a higher out-of-scanner response precision index regardless of trial type (r<−0.05, *P*<1.1×10^−02^) (Fig. 5e and Fig. S13), reaffirming its core function in timing^32^. Crucially, its in-task behavioural impact dynamically tracked temporal expectancy. In compressed trials (unpreparedness), higher readiness facilitated faster execution (Fig. 5e and Fig. S13) (r=−0.08, *P*=1.9×10^−4^). Conversely, in extended trials (lost temporal guidance), sustained high readiness paradoxically increased temporal instability, predicting both early and late errors (r>0.04, *P*<4.6×10^−2^) (Fig. 5e and Fig. S13). Mechanistically, this captures the dynamic allocation of vigilance: attention increases as the mental likelihood of the target accumulates, and would sustain after reaching the peak likelihood until the real target emerges^31,33,34^. Thus, prolonged cognitive readiness without temporal anchors eventually exhausts precision.

In sharp contrast, the cross-hemispheric discriminative signal showed no association with overall performance, i.e., the response precision index, across any temporal context (*P* > 0.05 for all conditions). Instead, it specifically predicted faster reaction times during consistent and extended trials (r<−0.04, *P*<4.1×10^−2^) (Fig. 5e and Fig. S13). Its failure to predict speed in compressed trials suggests that lateralised kinematic preparation is gated by, and secondary to, global cognitive readiness. Also, we observed similar double-dissociation patterns in the cerebellar motor areas (Fig. 5f and Fig. S13b).

Finally, mapping the brain-wide voxel contributions confirmed a profound and topologically stable spatial dichotomy. Strikingly, the spatial double dissociation observed in the IMAGEN dataset was perfectly replicated. Across temporal contexts, the hand-invariant shared component recruited a broad cortico-striato-thalamic network, encompassing the cognitive-only nodes of the SCAN and the bilateral putaminal fundus/thalamus (Fig. 5, Figs. S14 and S15). Meanwhile, the discriminative component remained strictly confined to classical effector-specific territories, actively bypassing the SCAN regions with negligible subcortical involvement (Fig. 5, Figs. S14 and S15).

### Parkinson’s genetic risk selectively impairs cognitive readiness

Impaired voluntary movement initiation is a hallmark of Parkinson’s disease (PD)^35^. Since major PD-associated genes also influence early neurodevelopment^36^, we tested whether PD polygenic risk scores (PD-PRS) perturb M1 preparatory signals. Longitudinal mixed-effects modelling across timepoints and conditions revealed that higher PD-PRS specifically predicted a reduced bi-hemispheric shared latent signal (β_std_=−0.058, *P*=1.4×10^−4^; Fig. 2j). Crucially, this impaired cognitive readiness mediated the association between PD-PRS and increased overall reaction time variability (overall RT SD) (proportion mediated=26.1%, *P*_bootstrap_=1.0×10^−4^, 95%CI=[0.0027, 0.0099]; Fig. 2j). In contrast, PD-PRS exhibited no association with the lateralised discriminative signal (β_std_=−0.008, *P*=0.32; Fig. 2j). This biological selective dissociation indicates that PD genetic risk preferentially blunts the global cognitive network for temporal precision rather than lateralised execution. Uncovering this deficit decades before clinical onset cross-validates our dual-system model and mechanistically explains why recent neuromodulation targeting the cognitive-only SCAN regions, rather than classical motor effectors, alleviates Parkinsonian symptoms^37^.

## Discussion

By decomposing bilateral preparatory signals across a 10-year longitudinal cohort and an independent cross-sectional cohort, we resolve a long-standing paradox in motor control and reveal a comprehensive brain-wide architecture of the mind-body interface. Our findings demonstrate that unilateral motor preparation is not a singular, contralateral relay event, nor is ipsilateral activity simply the byproduct of mirror-movement suppression or unspecific task effort. Rather, it requires the active, spatiotemporal intersection of two orthogonal systems superimposed within the primary motor cortex and cerebellum: a global, hand-invariant cognitive readiness network, and a highly lateralised, effector-specific kinematic preparation circuit. This activation-based spatial dichotomy is further corroborated by functional connectivity analyses (Fig. S16)^38^. This dual-system framework directly resolves the ipsilateral paradox by unifying seemingly contradictory models of interhemispheric cooperation and competition^4,5,14-16,39^. More importantly, it fundamentally subverts the classical, top-down hierarchy of motor control. M1 is not merely a passive downstream recipient of cognitive instructions from the prefrontal or supplementary motor areas. Instead, M1 operates as an independent, high-order cognitive hub that simultaneously computes and synchronises abstract intention with physical execution.

From an evolutionary and cellular perspective, this superimposed architecture is biologically imperative. An organism must be able to maintain and broadcast a global state of vigilance, a cognitive readiness, that can instantaneously trigger localised physical execution the moment a temporal threshold is met. Our macroscopic duality provides a human systems-level spatial architecture for the neuron population-level mathematical subspaces observed in non-human primates^6,18,40^, perfectly mirroring the microscopic division of labour observed in layer 5 cortical projection neurons^8,9^. The bilaterally shared component acts as the macroscopic equivalent of the output-null subspace. Encompassing the newly defined somato-cognitive action network (SCAN) (Fig. 3)^10^ and putaminal fundus, this network maps onto dominant, movement-invariant Layer 5 population dynamics^9^. Broadcast transcallosally via IT neurons, this global intention is likely sustained by PTupper neurons forming robust cortico-ponto-cerebellar loops that return via the ventrolateral thalamus, establishing a unified readiness state without leaking premature execution signals, potentially through maintaining a temporal threshold^8,12^. Conversely, the lateralised discriminative component provides the spatial footprint of the output-potent subspace. Here, movement-specific preparation, initially encoded by localised IT sub-populations, funnels into lateralised PT_lower_ output tracts to drive the descending commands for execution once the global timing gate opens.

Remarkably, this cognitive-kinematic dichotomy fundamentally extends to the cerebellum, revealing that voluntary movement relies on a continuous, three-stage mind-body synchronisation across the cortico-cerebellar axis. First, during intention, M1 acts as the initial integrative hub, structurally superimposing the globally broadcast cognitive readiness state with the highly localised kinematic preparation plan^1,2^. Second, for timing to initiate, these parallel signals descend to the cerebellum, which acts as a crucial coincidence detector^12,13^. By computing the precise temporal intersection of the widespread intention network and the localised kinematic loops, the cerebellum determines the exact moment of initiation^12,13^. Third, during execution, the cerebellum broadcasts this temporal decision back to M1 via the ventrolateral thalamus^8,12^. This ascending pulse triggers a final, definitive synchronisation within M1—opening the temporal gate, shifting the cortical population into an output-potent state^6,7^, and locking the cognitive 'go' signal to the descending physical execution command. Thus, at every critical juncture of movement, the central nervous system enforces a strict, active synchronisation between cognitive intention and kinematic execution.

Crucially, this framework redefines the functional role of the motor system in generalised timing and temporal expectation, offering profound clinical insights into neurodegenerative conditions like Parkinson’s disease (PD). PD has long been clinically linked to early deficits in motor timing, temporal discrimination, and attenuated readiness potentials in higher-order motor areas^41-43^. By demonstrating that Parkinson’s genetic loading selectively blunts the bi-hemispheric shared cognitive readiness signal in a healthy adolescent cohort and directly mediates the genetic risk’s negative impact on temporal precision, we provide critical evidence that this vulnerability manifests decades before the onset of classic kinematic symptoms. Mechanistically, this indicates that the genetic burden of PD fundamentally disrupts the globally broadcast temporal intention network, including the subcortical putaminal fundus and the M1-spanning SCAN, while initially sparing the lateralised execution circuits. This early functional disruption aligns perfectly with recent evidence that many classic late-onset PD genes are actually highly expressed during embryonic and postnatal brain development^44^, explaining how these distinct cognitive-motor developmental deficits can precede typical neurodegeneration.

Ultimately, this dual-process framework provides a biological blueprint for how the human brain integrates abstract thought with physical embodiment. Clinically, this dual process model suggests principled therapeutic strategies: targeted treatments could restore discriminative drive for specific kinematic deficits (e.g., hemiparesis), whereas neuromodulation of the broadly cognitive readiness network, for instance, SCAN, could stabilise temporal precision and initiation deficits in early-stage Parkinson’s disease^35,37^. Furthermore, beyond motor control, our latent signal decomposition framework provides a generalisable analytical paradigm for broad cognitive neuroscience. Many higher-order human functions, such as language, spatial attention, and working memory, rely on highly lateralised core regions coupled with broader bilateral engagement. By mathematically decoupling interhemispheric cooperation (shared global states) from interhemispheric competition (lateralised specific processing), future research can dissect the hidden mind-body or global-local architectures across the entire human cerebral cortex.

## Methods

### Participants

More than a thousand adolescents from the IMAGEN project were included in our study^20,21^. Behaviour and fMRI data were collected from 8 sites across France, the United Kingdom, Ireland, and Germany. The research project was approved by ethics committees, with informed consent obtained from participants and their guardians. The research protocol and procedures of data acquisition have been published^45^. Our study examined data of right-handed individuals at baseline (BL, age 14, N=1601), follow-up 2 (FU2, age 19, N=1143), and follow-up 3 (FU3, age 23, N=953).

### Monetary incentive delay (MID) task

Participants completed a modified version of the MID task to investigate neural responses to reward anticipation and outcome^20,21^. The task comprised 66 trials that lasted 10 seconds each. In each trial, participants were presented with one of three cue shapes (250 ms) indicating the target's location on the left or right side of the screen and the potential reward of 0, 2, or 10 points. After a delay of 4000 to 4500 ms, participants were required to respond with a left or right button press (with the corresponding left or right hand) as soon as the target appeared. Feedback on the trial outcome was displayed for 1450 ms after the response, indicating the number of points won. Using a tracking algorithm, task difficulty was adjusted for each participant, ensuring they responded successfully to around 66% of trials. Before the scan, participants completed a practice session (~5 min) and were informed that for every five points won, they would receive a small chocolate snack at age 14 or a small amount of cash at age 19 or 23.

### MRI data acquisition

Neuroimaging data were collected across eight independent assessment sites utilising 3T MRI platforms from four major vendors (Philips, Siemens, Bruker, and General Electric). To minimise multi-site variance, a universally compatible acquisition protocol was strictly harmonised across all scanners. For anatomical reference, high-resolution three-dimensional T1-weighted structural images (1.1 mm^3^ isotropic voxels) were obtained utilising a gradient-echo sequence adapted from the Alzheimer’s Disease Neuroimaging Initiative (ADNI) guidelines. Task-based functional imaging was conducted using a blood-oxygen-level-dependent (BOLD) gradient-echo echo-planar imaging (EPI) sequence. The functional acquisition parameters were defined as follows: repetition time (TR)=2200 ms, and echo time (TE)=30 ms. Each task fMRI run comprised 300 volumes, with every volume containing 40 slices oriented parallel to the anterior commissure-posterior commissure (AC-PC) axis (slice thickness=2.4 mm, inter-slice gap=1 mm).

### Task-based functional image preprocessing

Preprocessing of the functional datasets was executed via the fMRIPrep pipeline (version 20.2.3) ^46^. The spatial normalisation workflow commenced with the generation and skull-stripping of a representative BOLD reference volume. To account for susceptibility-derived artefacts, fMRIPrep's fieldmap-less approach was applied to estimate a deformation field, which was subsequently aligned to the functional reference. This distortion-corrected functional reference was then precisely co-registered to the corresponding T1-weighted anatomical image utilising boundary-based registration (BBR). Following rigorous adjustment for head motion and slice-timing correction, the resulting BOLD time-series were non-linearly transformed into the standard MNI coordinate space. After the removal of linear trends from these normalised time series, the preprocessed functional data were resampled to a resolution of 3×3×3 mm^3^ and spatially smoothed utilising a 6-mm full-width at half-maximum (FWHM) Gaussian kernel.

### Activation of task-based fMRI

First-level analysis was conducted utilising the Statistical Parametric Mapping software package (SPM; http://www.fil.ion.ucl.ac.uk/spm). At the first-level analysis, the BOLD signal for each subject was assessed by a linear combination of regressors for experimental conditions, i.e., outcomes (hit and miss)×phases (anticipation, pressing, and feedback)×reward magnitudes (No-win, Small-win, and Big-win)×hands (left and right), where the regressor modelling each of the experimental conditions was established by convolving the corresponding experimental condition with SPM’s canonical hemodynamic response function. The final general linear model (GLM) contained 36 task-condition regressors and 26 additional covariates. These covariates included the mean signals extracted from the white matter and ventricles, combined with a 24-parameter head motion model (6 rigid-body realignment parameters, their first-order temporal derivatives, and the squared values of all 12 terms). These regressors were used with the SPM to estimate the condition-specific brain signal, i.e., the regression coefficient of the corresponding task-condition regressor in the GLM.

Intersections of voxels with significant activation (p_FWE_<0.05) across timepoints (BL, FU2 and FU3) and reward conditions were used as ROIs for calculating hand-related activation, which were located in the contralateral hand-knob area in the M1. The correlations of the hand-related signals and the reaction time were calculated to evaluate the extent to which the hand-related activation could explain the reaction time. Also, multiple linear regression (MLR) models were employed to determine the contribution of different hand-related signals (e.g., across different task conditions and left/right trials) for reaction time.

### Signal decomposition

We hypothesised that the fMRI signals detected in bilateral hand-related areas during the task consisted of two components: a shared latent signal and a discriminative latent signal, which remained the same regardless of the hand used. The latent component signals were calculated using the multiple linear model, which was applied to the four activation signals (signals in the bilateral hand-related areas when either hand was used) of each participant, with (1, 1) (for contralateral signal) and (1,−1) (for ipsilateral signal) as independent variables to model the coefficients as the decomposed signals, which were assumed to remain the same across hands.


{Contralateral Signal=Latent Shared Signal+Latent Discriminative SignalIpsilateral Signal=Latent Shared Signal−Latent Discriminative Signal}

### Voxel-wise contribution mapping (fixed-effects MLR)

We quantified the brain-wide associations with the shared and discriminative latent signals using separate MLR models at each voxel v, fitted independently for each condition (big/small/no win; left/right hand).

For a given condition, the dependent variable Yv (the first-level beta value stacked across observations) and the two continuous latent predictors were first Z-scored. The design included these standardised continuous predictors along with unstandardized categorical fixed effects for session and subject (participant indicators), i.e., a subject fixed-effects specification (no random effects). To ensure identifiability, one level per categorical factor was omitted (reference coding).

The voxel-wise model was:

$$Y_{v}=\beta_{o,v}+\beta_{lat,v}D+\beta_{sha,v}S+d_{v}^{T}X_{session}+s_{v}^{T}X_{subject}+\varepsilon_{v}$$

where D and S denote the standardised condition-specific discriminative and shared latent signals, X_session_ are 2 timepoint indicators, and X_subject_ are subject dummies. Models were estimated by MLR with an intercept; coefficient t-statistics and two-sided p-values were retained.

We reassembled β_lat,v_ and β_sha,v_ into 3D images in MNI space to obtain voxel contribution maps for the discriminative and shared components, respectively; the corresponding p-value maps were generated from the MLR t-tests. Positive coefficients indicate a greater voxel contribution to the corresponding latent signal after controlling for session and subject fixed effects.

### Linear mixed-effects modelling of genotype-phenotype associations

To investigate the genomic basis of the identified motor regulatory mechanisms, we examined the association between the Parkinson's disease polygenic risk score (PD-PRS) and the derived latent motor signals (shared and discriminative). The PD-PRS was calculated utilizing base summary statistics from a large-scale genome-wide association meta-analysis^36^. Following standard variant-level quality control (excluding SNPs with >5% missingness), missing genotypes at the individual level were handled via mean allele frequency imputation^47,48^. Given the hierarchical nature of our dataset—which includes repeated measures across three longitudinal timepoints (Baseline, Follow-up 2, and Follow-up 3) and three experimental conditions (Big-win, Small-win, and No-win)—we employed a Linear Mixed-Effects Model (LMM) to maximise statistical power and capture stable, trait-like associations robust to state-dependent fluctuations. For each model, the latent fMRI signal was treated as the dependent variable, with PD-PRS as the primary fixed effect of interest. To rigorously control for potential confounds and population stratification, we included age, sex, imaging site, task condition, and study timepoint as fixed covariates, along with the top ten genomic principal components (PCs). A random intercept was included for each participant to account for within-subject correlations inherent in the repeated-measures design. All continuous variables were z-standardised prior to model fitting to facilitate the interpretation of standardised regression coefficients (β).

### Multilevel mediation analysis

We conducted a multilevel mediation analysis to test the mechanistic hypothesis that the latent signals mediate the impact of PD genetic risk on task performance. Leveraging the LMM framework described above, we constructed a causal path model connecting the predictor (PD-PRS), the mediator (latent signal), and the outcome (overall mean RT SD). The analysis involved estimating three specific pathways: the effect of genetic risk on the neural mediator (Path a); the effect of the neural mediator on behavioural performance while controlling for genetic risk (Path b); and the direct effect of genetic risk on performance (Path c'). The total effect (Path c) of PD-PRS on performance was also estimated. The statistical significance of the indirect effect (the product of coefficients a×b) was assessed using a bootstrapping procedure with 10,000 iterations. Significance was determined based on whether the 95% bias-corrected confidence interval (CI) of the indirect effect excluded zero.

### Zhangjiang International Brain BioBank (ZIB) cohort

To assess the generalisability of our findings, we utilised the college cohort from Zhangjiang International Brain BioBank (ZIB, N=2,638, https://zib.fudan.edu.cn/), where participants performed a modified version of the MID task adapted from the ABCD study^32^. Unlike IMAGEN, this task involved unimanual responses and highly variable anticipation durations (1,500–4,000 ms). To strictly control for sequential effects, we classified trials based on the duration difference (Δ) between the current and preceding trials.

This stratification yielded three distinct categories: extended trials (Δ > 1s; current > previous), stable trials (0 ≤ Δ ≤ 1s), and compressed trials (Δ<0s; current<previous). Differentiation was necessary because these contexts impose distinct temporal expectations that differentially modulate preparatory motor states. Finally, to maximise the signal-to-noise ratio given the limited number of trials per condition, we combined the data across all 5 reward valences to isolate the generic signal.

Notably, outside the MRI scanner, each participant also conducted a practice session of the MID task, with a fixed delay duration of 2,000 ms. The hence derived 95% upper boundary of RT (calculated as the mean RT plus two standard deviations of the reaction time, and used to calibrate the initial task difficulty in the scanner^32^) or the precision index (i.e., a smaller 95% upper boundary of RT indicates a higher precision in response for a given time interval) was also included in our analysis to serve as a baseline reference for response, indexing intrinsic psychomotor capacity when a target could be accurately predicted (i.e., without temporal variability in delay duration).

To extract the latent motor regulatory signals in the ZIB cohort, we employed Principal Component Analysis (PCA) rather than the previous model-based orthogonal decomposition. This shift is necessary because the ZIB task adopted a unimanual response paradigm, lacking the left-right hand alternation required to maintain the structural symmetry of the predefined orthogonal model. Since our IMAGEN data already demonstrated that the first two PCs perfectly replicate the shared and discriminative latent components (r > 0.99), PCA serves as a robust, data-driven alternative to isolate the dual-system signals in this unbalanced design.

### Task-based functional connectivity analysis

To delineate the network-level topologies driven by the two distinct motor preparation processes, task-based functional connectivity (FC) was computed using the CONN toolbox (version 16a) integrated with SPM12^49^. The first-level General Linear Model SPM.mat design files previously generated for the activation analyses were directly imported, ensuring that all condition-specific temporal parameters, task regressors (e.g., the anticipation phase of Baseline Big-win successful trials), and motion covariates were strictly preserved. Seed-to-voxel bivariate correlations were estimated utilising a standard HRF-weighted model. Crucially, to isolate the discrete connectivity profiles of the cognitive readiness and kinematic preparation systems, the extracted BOLD signals from the bilateral motor ROIs were orthogonally decomposed into shared and discriminative seed time series prior to FC estimation, using a spatial signal-splitting framework established in our previous work^38^. The resulting connectivity maps capture the independent brain-wide synchronisation patterns of the hand-invariant and hand-specific latent signals.

## Supplementary Material

1

Supplementary Information is available for this paper. Correspondence and requests for materials should be addressed to Tianye Jia. Reprints and permissions information is available at www.nature.com/reprints.

## Figures and Tables

**Fig. 1.**
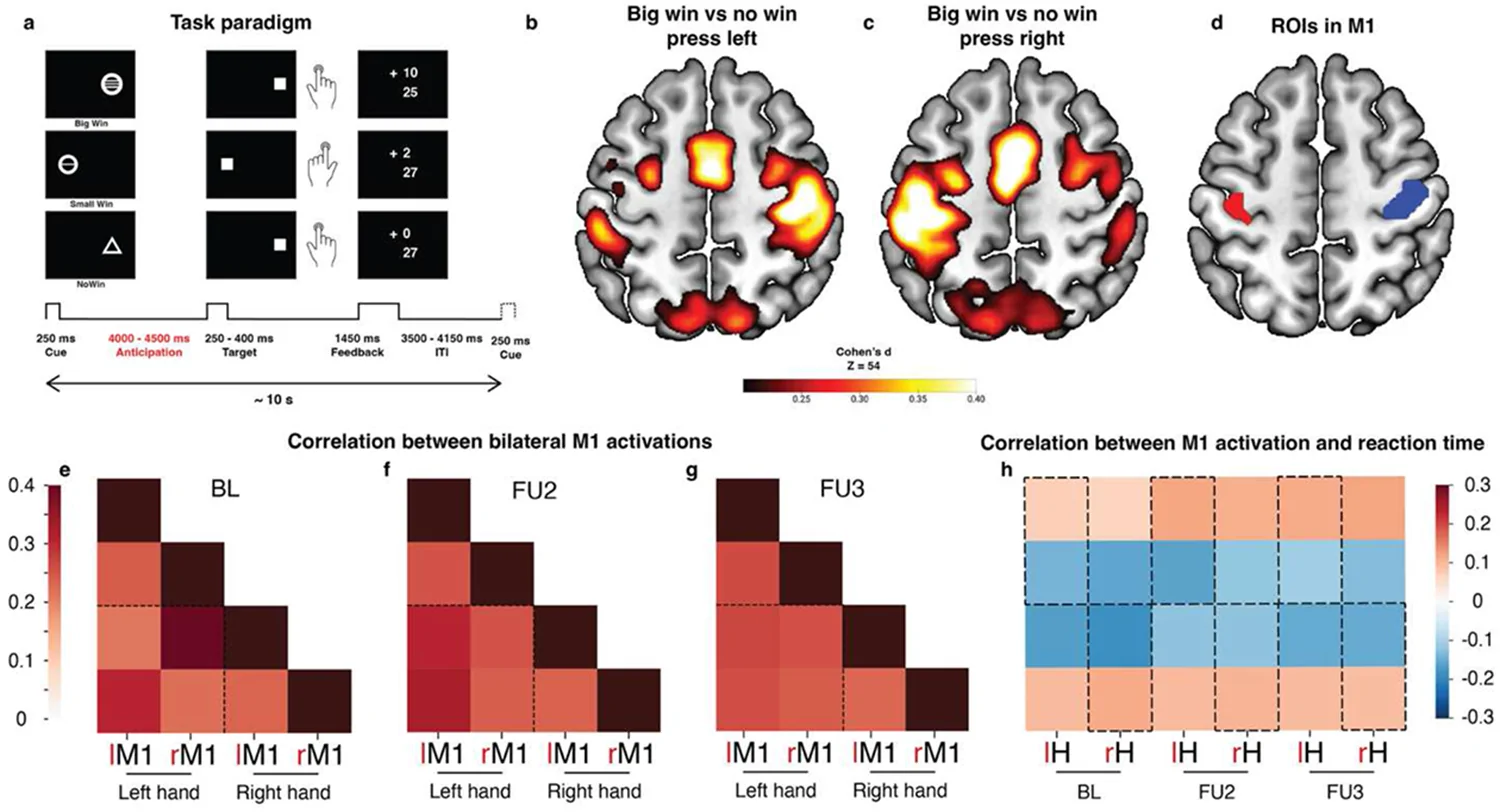
Bilateral M1 engagement during unimanual motor preparation demonstrates opposing associations with reaction time. (**a**) Paradigm of the modified Monetary Incentive Delay (MID) task. Each trial begins with a cue (250 ms) indicating potential reward magnitude and the target hand. During the anticipation phase (~4000 to 4500 ms), participants prepare for a unimanual response. (**b** and **c**) Activation maps comparing Big Win versus No Win anticipation phases for left-hand and right-hand responses at Baseline (BL). Bilateral activations are observed in the M1 despite the unimanual task requirement. (**d**) Regions of interest (ROIs) in the bilateral M1 hand-knob area, representing the intersection of significant voxel-wise activations (*P*_FWE_<0.05) across conditions and timepoints [BL, Follow-up 2 (FU2), Follow-up 3 (FU3)]. (**e** to **g**) Correlation matrices of bilateral M1 activations (left M1, lM1; right M1, rM1) during the Big Win condition across responding hands at BL (E), FU2 (F), and FU3 (G). Contralateral and ipsilateral activations exhibit strong positive coupling. (**h**) Correlation between bilateral M1 activations and reaction time (RT) for successful Big Win trials. Despite their positive co-activation, contralateral and ipsilateral M1 signals exhibit opposing behavioural associations: contralateral M1 negatively correlates with RT, whereas ipsilateral M1 positively correlates with RT.

**Fig. 2.**
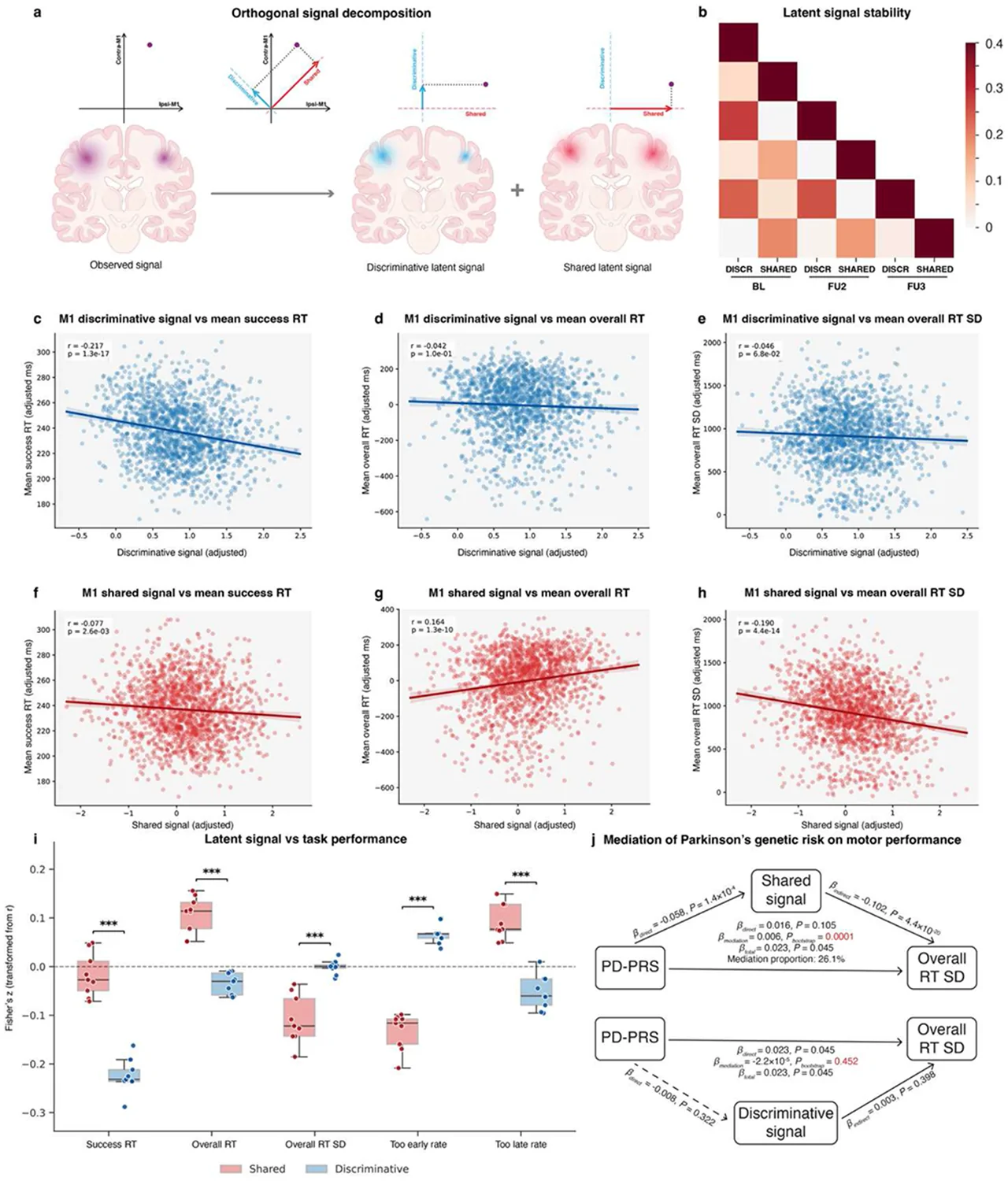
Orthogonal decomposition of bilateral M1 signals reveals a double dissociation in motor regulation and mediates Parkinson's disease genetic risk. (**a**) Schematic of M1 signal decomposition into a hand-invariant shared signal (red) and a cross-hemispheric discriminative signal (blue). (**b**) Correlation matrix across longitudinal timepoints confirming intra-component stability and inter-component orthogonality. (**c** to **h**) Scatter plots linking baseline latent signals to behaviour. Discriminative signals specifically predict successful execution speed (c), but remain uncoupled from global timing (d and e). Conversely, shared signals govern global response precision, correlating strongly with overall RT (g) and variability (h), while showing weak associations with success RT (f). Solid lines indicate linear fits with 95% confidence bands. (**i**) Correlation summaries confirming a double dissociation: discriminative signals exclusively regulate execution speed, whereas shared signals regulate global temporal precision and vigilance. (**j**) Multilevel mediation analysis linking Parkinson's disease polygenic risk score (PD-PRS) to motor performance. Higher PD-PRS attenuates the shared signal, sequentially mediating the genetic risk's negative impact on overall RT SD (top); no parallel association is observed for the discriminative signal (bottom).

**Fig. 3.**
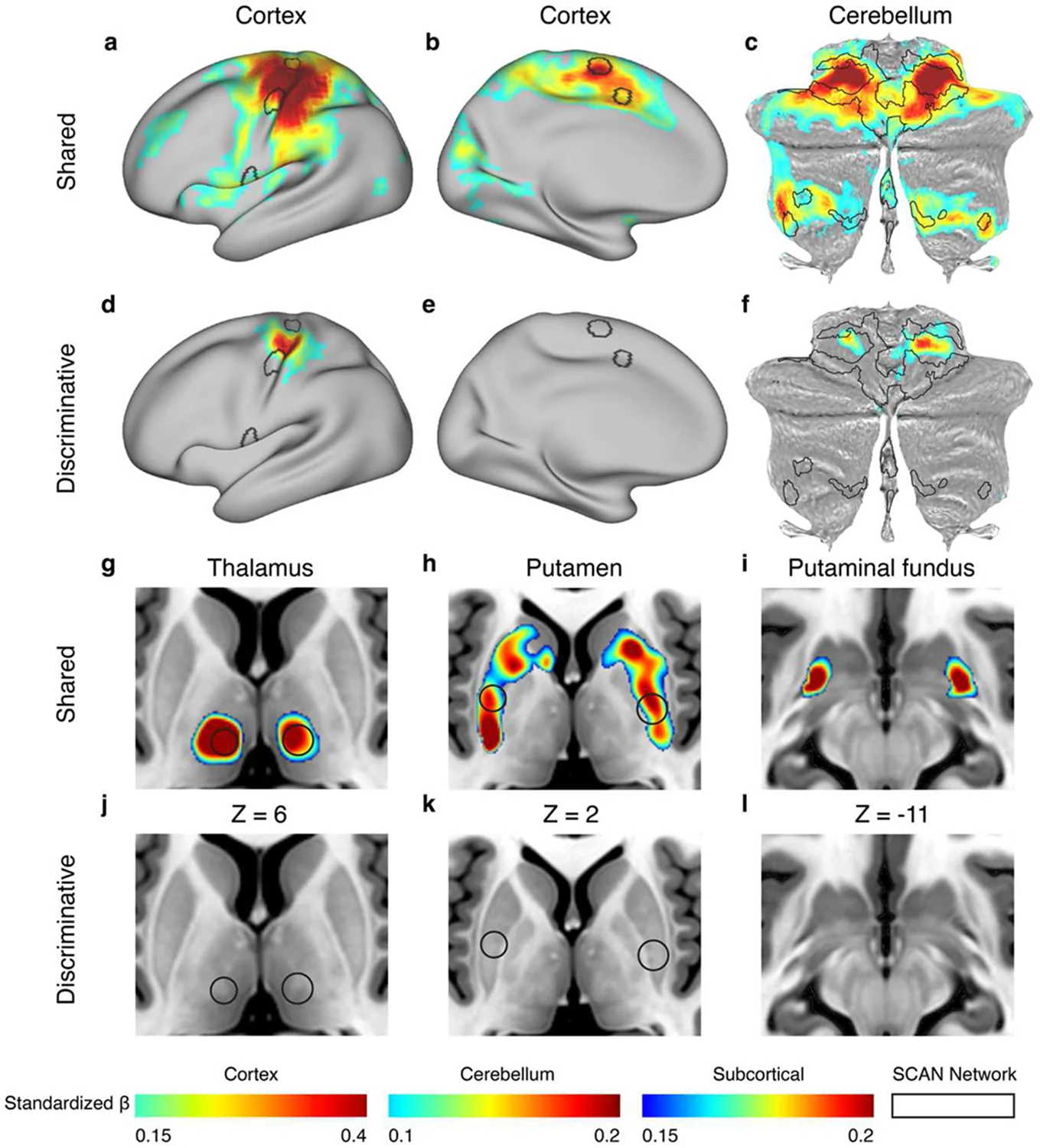
Brain-wide anatomical dissociation of cognitive readiness and kinematic preparation networks. (**a** to **c**) Brain-wide maps of the hand-invariant shared signal (cognitive readiness) across the cerebral cortex (a and b) and cerebellum (c). This signal exhibits broad, bilateral recruitment, strongly matching the a priori defined Somato-Cognitive Action Network (SCAN) regions (black outlines). (**d** to **f**) Maps of the discriminative signal (kinematic preparation). Unlike the shared signal, it localises to classical effector-specific motor territories (e.g., M1 hand-knob area) and spares SCAN regions. Panel (f) overlays hand-specific discriminative signals from both left- and right-hand responses; other panels reflect right-hand activation. (**g** to **l**) Subcortical mapping reveals profound structural dissociation. The shared signal (g to i) robustly recruits the central cortico-striato-thalamic loop, engaging the thalamus (g), putamen (h), and putaminal fundus (i). Conversely, the discriminative signal (j to l) minimally engages these subcortical areas. Subcortical statistical maps are masked to anatomical boundaries.

**Fig. 4.**
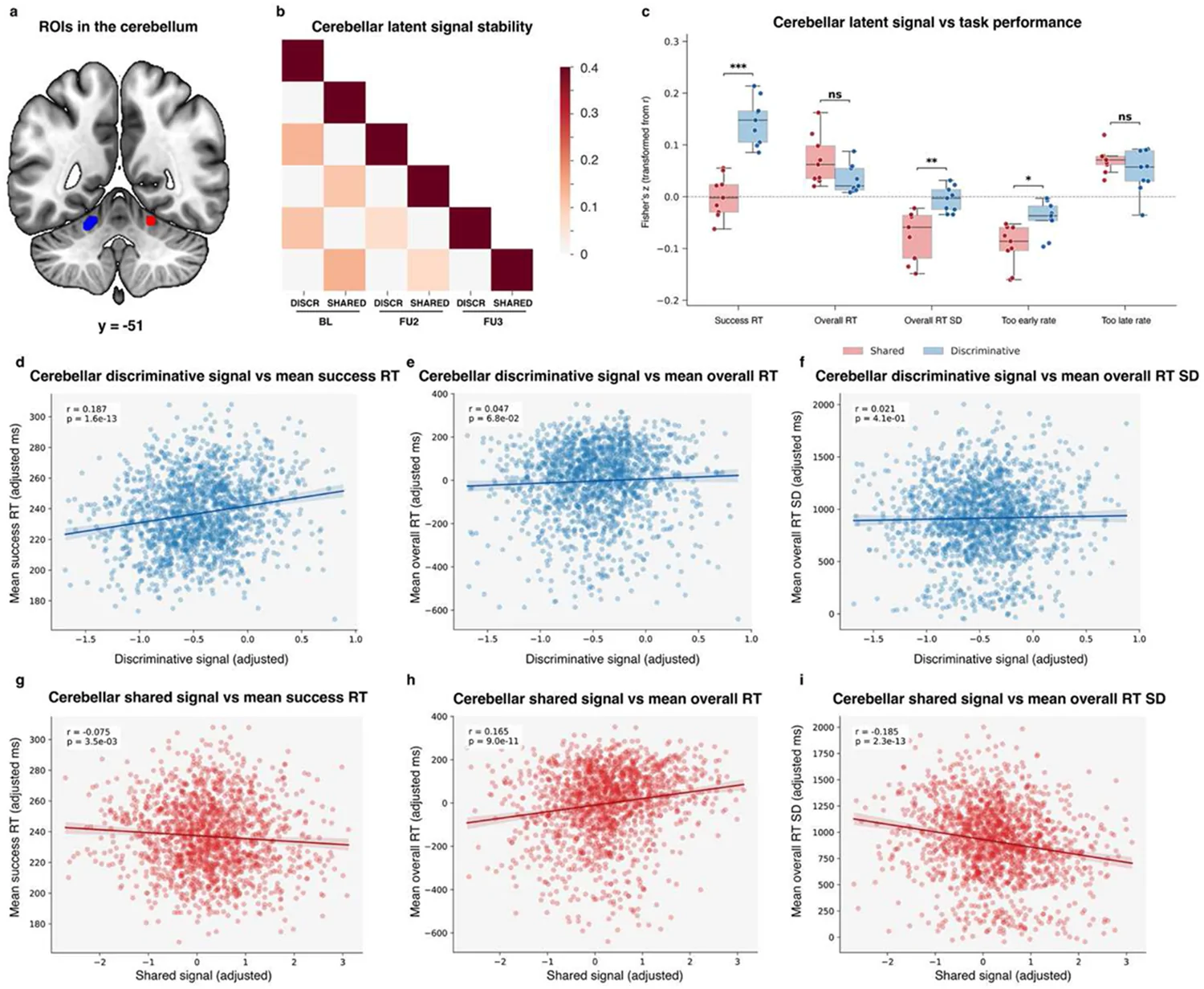
Corroboration of the cognitive-motor dual system in the cerebellum. (**a**) Anatomical localisation of the functionally defined cerebellar ROIs. (**b**) Correlation matrix of the decomposed shared and discriminative signals extracted from the cerebellar hand areas. Mirroring the patterns in M1, the cerebellar latent signals demonstrate robust longitudinal stability and inter-component orthogonality. (**c**) Summary of behavioural associations across all timepoints and task conditions for the cerebellar signals. Boxplots of Fisher's z-transformed correlation coefficients confirm that the double dissociation observed in M1 is preserved in the cerebellum. (**d** to **i**) Representative scatter plots (Baseline, Big Win) illustrating the distinct behavioural footprints of the cerebellar latent signals. The cerebellar discriminative signal specifically predicts execution speed (Success RT, d) but not global metrics (e and f). In contrast, the cerebellar shared signal governs overall temporal precision, correlating with Mean Overall RT (h) and Overall RT SD (i).

**Fig. 5.**
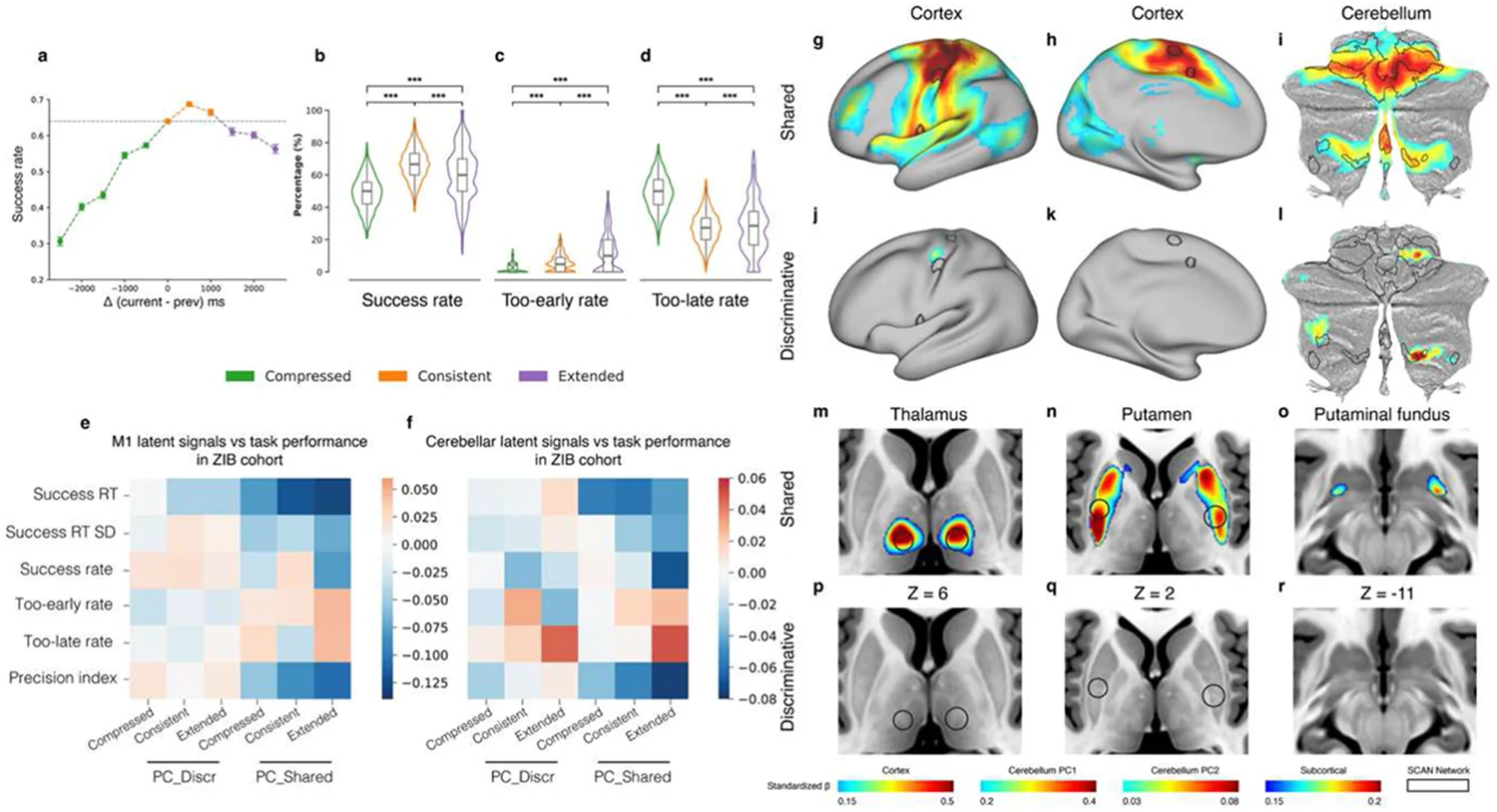
Generalisability of the cognitive-motor dual system and spatial dissociation across temporal contexts in the ZIB cohort. (**a**) Success rate across inter-trial delay differences (Δ), categorising trials into compressed (Δ<0 ms), consistent (0≤Δ≤1000 ms), and extended (Δ>1000 ms) contexts. (**b** to **d**) Violin plots of Success (b), Too-Early (c), and Too-Late (d) rates across contexts. Compressed trials exhibit severe late-error impairments; extended trials show bidirectional precision loss via premature and delayed errors (***P<0.001). (**e** and **f**) Correlations linking M1 (e) and cerebellar (f) latent signals to behaviour. The double dissociation is preserved: the shared signal regulates global precision and context-dependent vigilance, while the discriminative signal selectively facilitates execution speed. (**g** to **r**) Voxel contribution maps of shared (cognitive readiness; g to i, m to o) and discriminative (kinematic preparation; j to l, p to r) signals. Replicating IMAGEN findings, the shared signal recruits bilateral cortices overlapping the Somato-Cognitive Action Network (SCAN, black outlines), cerebellar hand areas (i), thalamus (m), and putamen (n and o). The discriminative signal strictly localises to effector-specific regions, sparing SCAN (j and k) with negligible subcortical engagement (p to r). Subcortical statistical maps are masked to anatomical boundaries.

## Data Availability

The IMAGEN data are available from a dedicated database: https://imagen-project.org. The ZIBdata are available from a dedicated database: https://zib.fudan.edu.cn/.

## Code availability

All scripts and codes used to generate our results are publicly accessible through a GitHub repository (https://github.com/c2heng/cognitive_motor_dual_model/).
