# Supplementary material for: Cognitive-Motor Duality in Human M1 and Cerebellum
Source: Res Sq. 2026 Sep 10:rs.3.rs-10164419. Preprint. [Version 1] doi: 10.21203/rs.3.rs-10164419/v1 (PMC13622857; doi:10.21203/rs.3.rs-10164419/v1)
Supplement: Supplementary file 1 [file NIHPPrs10164419v1-supplement-1.pdf]

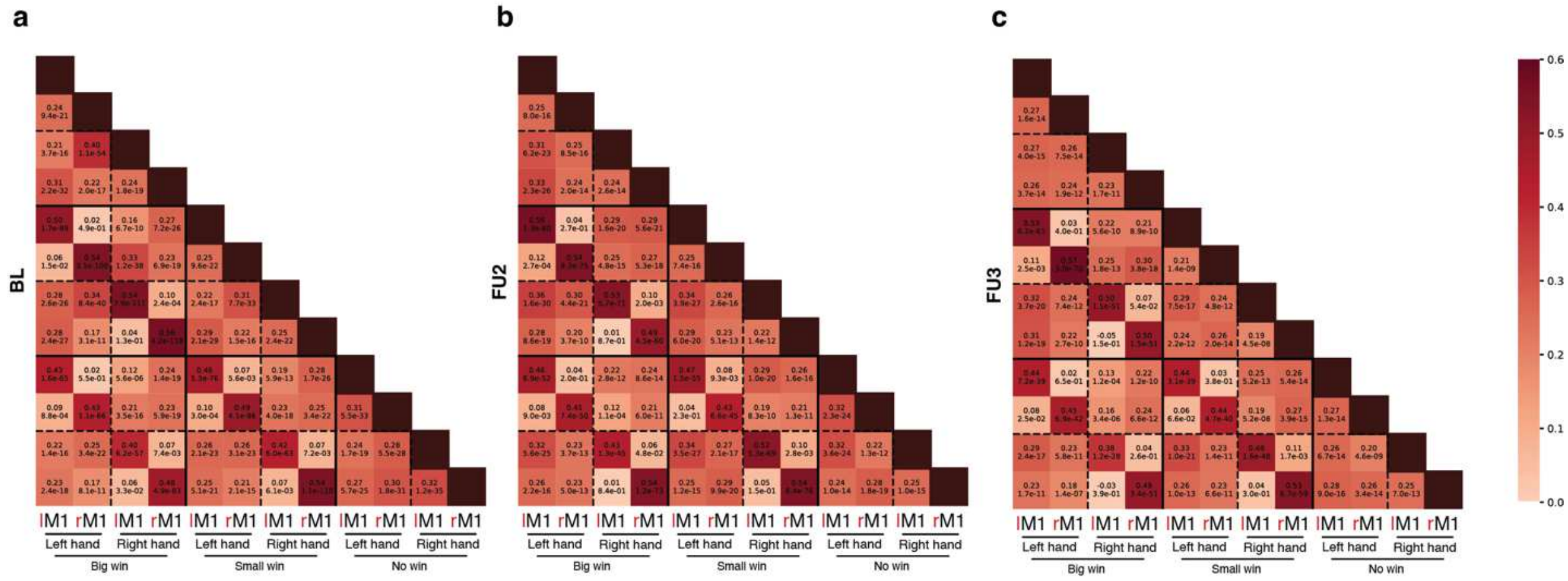

Fig. S1. Correlation matrices of bilateral M1 activations across all longitudinal timepoints. Detailed triangular heatmaps displaying the correlation between left (lM1) and right (rM1) primary motor cortex activations during the anticipation phase. Data are presented separately for the Baseline (BL, **a**), Follow-up 2 (FU2, **b**), and Follow-up 3 (FU3, **c**) sessions. The axes denote all combinations of responding hands (Left Hand, Right Hand) and reward contexts (Big Win, Small Win, No Win). The color gradient indicates the strength of the positive correlation (Pearson's r). Within each cell, the exact correlation coefficient r is annotated at the top, with its corresponding P value annotated at the bottom.

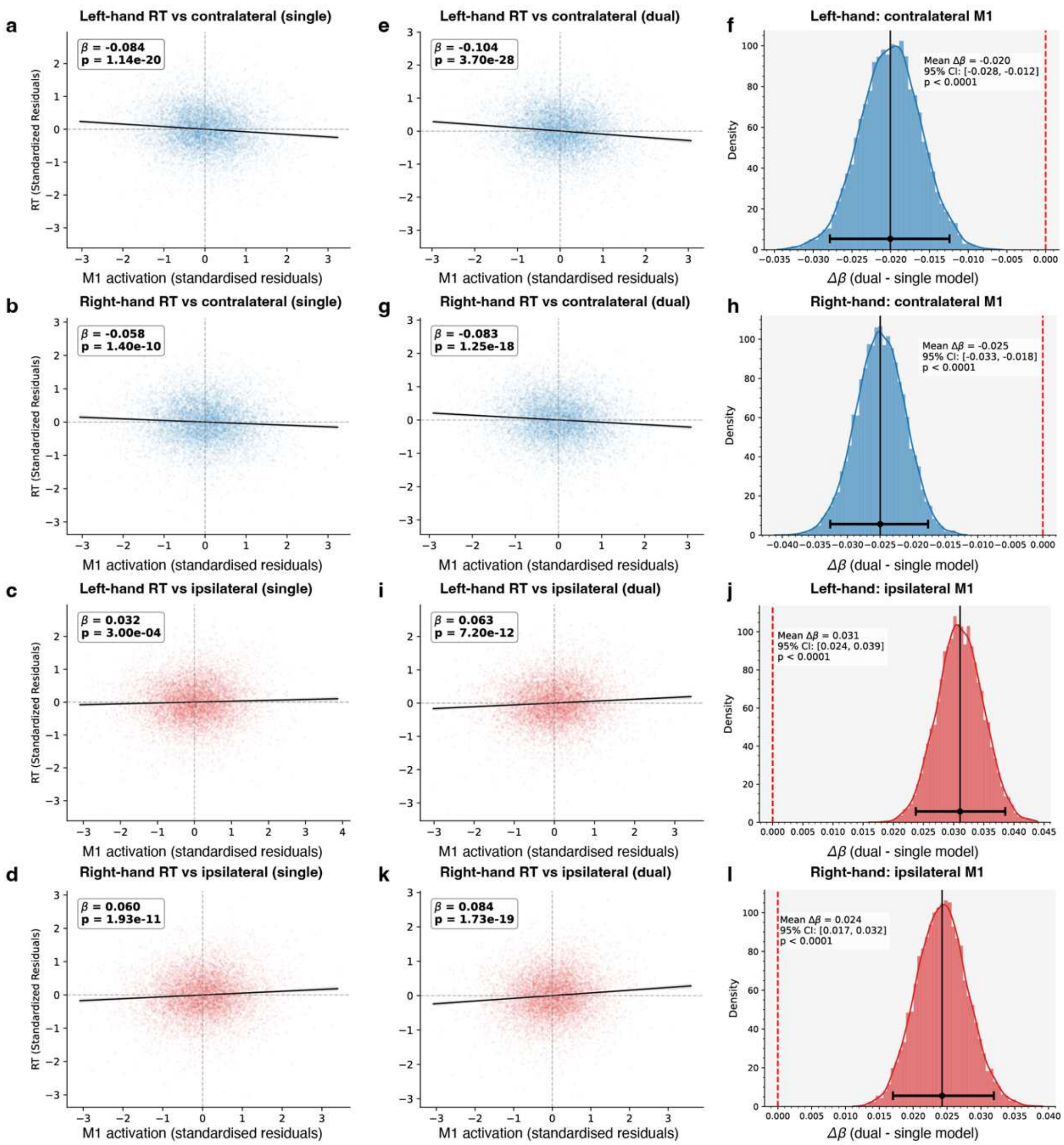

Fig. S2. Mutual enhancement of behavioural associations in bilateral M1 reveals a statistical suppression effect. (**a** to **d**) Linear mixed-effects model (LMM) associations between reaction times (RT) and M1 activations when hemispheres are modelled independently (Single model). Contralateral M1 signals (**a** and **b**; blue) show a significant negative association with RT, whereas ipsilateral signals (**c** and **d**; red) exhibit a positive association for both left- and right-hand responses. (**e**, **g**, **i**, and **k**) Scatter plots demonstrating the strengthened behavioural associations when bilateral M1 signals are simultaneously included in the LMM (Dual model), mutually

controlling for one another. (**f**, **h**, **j**, and **l**) Bootstrapped distributions of the difference in standardised coefficients ($\Delta\beta$) between the Dual and Single models (i.e., Dual minus Single). The shifts in $\Delta\beta$, becoming more negative for contralateral M1 (**f** and **h**) and more positive for ipsilateral M1 (**j** and **l**), confirm that controlling for the opposite hemisphere significantly amplifies the lateralised functional contributions to execution speed. Solid black lines indicate the 95% confidence intervals, and red dashed lines denote the null hypothesis ($\Delta\beta$=0).

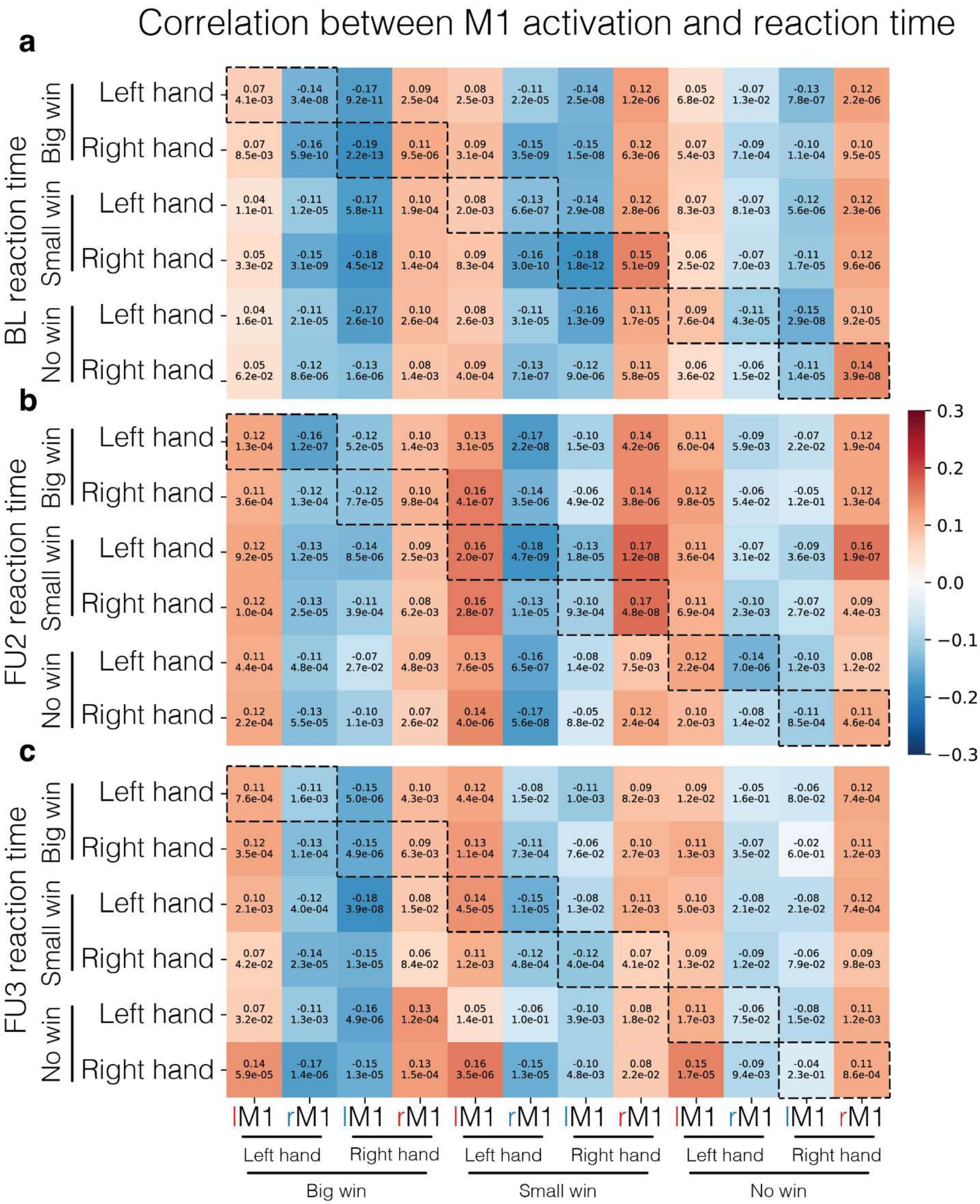

Fig. S3. Robust and consistent associations between bilateral M1 activations and reaction times across all task conditions and longitudinal timepoints. Detailed heatmaps displaying the

partial correlations between primary motor cortex (lM1, rM1) activations and behavioural reaction times (RT) at Baseline (BL, **a**), Follow-up 2 (FU2, **b**), and Follow-up 3 (FU3, **c**). The axes represent all combinations of responding hands (Left Hand, Right Hand) and reward contexts (Big Win, Small Win, No Win). The colour gradient reflects the strength and direction of the correlation, with exact correlation coefficients and corresponding P values annotated at the top and bottom of each cell, respectively. Dashed black boxes highlight the associations within the exact matched task conditions. All correlations between a specific M1 activation and RT were calculated after controlling for the activation of the counter-hemisphere. Consistent with the aggregated model, the matrices reveal a strict lateralised dichotomy: contralateral M1 activations consistently show strong negative correlations with RT (blue cells; facilitating faster responses), whereas ipsilateral M1 activations exhibit positive correlations with RT (red cells; associated with slower responses). This robust pattern is highly preserved across every single timepoint, reward condition, and responding hand configuration. Values within cells denote Pearson's r and their corresponding P values.

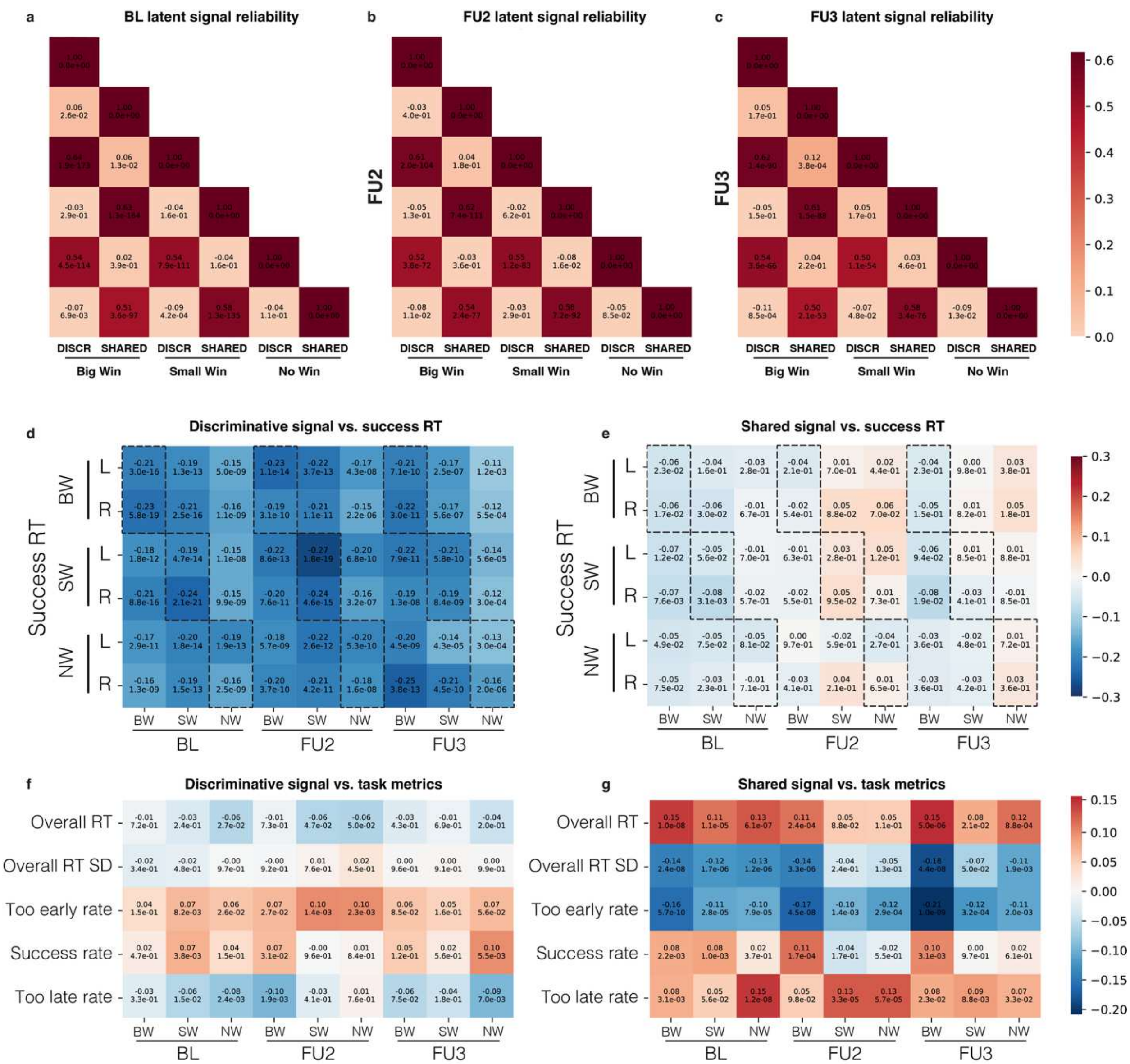

Fig. S4. Comprehensive evaluation of latent signals' reliability and their dissociable behavioural associations across all timepoints. (**a** to **c**) Correlation matrices of the decomposed discriminative (DISCR) and shared latent signals within Baseline (**a**, BL), Follow-up 2 (**b**, FU2), and Follow-up 3 (**c**, FU3). The patterns reveal strong intra-class correlations among the same signal types across different reward conditions, accompanied by near-zero inter-class correlations between the discriminative and shared signals. This consistently validates the orthogonality of the signal decomposition at every longitudinal stage. (**d** and **e**) Detailed heatmaps displaying the exact

correlations between success reaction time (RT) and the discriminative (**d**) and shared (**e**) latent signals across all combinations of responding hands and reward contexts. Dashed black boxes highlight the associations within matched task conditions. The discriminative signal robustly and selectively predicts faster execution speed (negative correlations; blue cells) in successful trials, a relationship absent in the shared signal. (**f** and **g**) Heatmaps detailing the relationships between the latent signals and a broader global task performance metric. Demonstrating a strict double dissociation, the shared signal (**g**) exhibits highly consistent associations with metrics of cognitive timing and precision (e.g., concentrated response patterns indicated by lower RT standard deviation and lower too-early rates), whereas the discriminative signal (**f**) remains unassociated with these metrics. Values within cells denote Pearson's r and their corresponding P values.

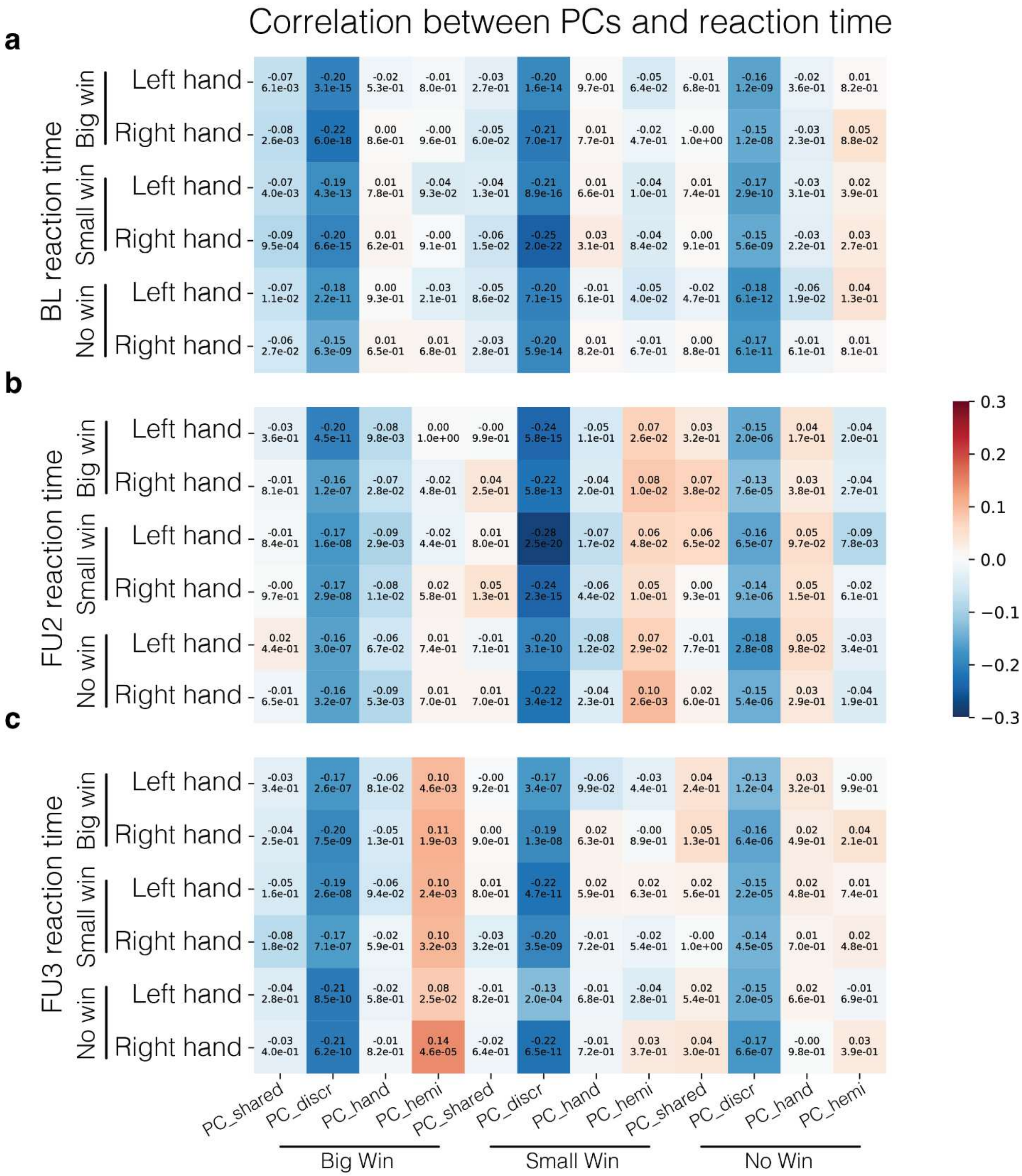

Fig. S5. Associations between model-free principal components and successful reaction time across longitudinal timepoints. (**a** to **c**) Heatmaps displaying the Pearson correlation coefficients (r, top values in each cell) and corresponding P values (bottom values) between the four principal

components (PCs) extracted via model-free PCA (PC_Shared, PC_Discr, PC_Hand, PC_Hemi) and the reaction time (RT) of successful trials at Baseline (**a**), Follow-up 2 (**b**), and Follow-up 3 (**c**). Within each timepoint panel, the correlation matrices are systematically stratified by the task reward conditions during which RT was recorded (Big Win, Small Win, No Win), the responding hand (rows), and the task conditions from which the PCs were originally derived (column blocks). Consistent with the model-based signal decomposition (Fig. 2), PC_Discr (capturing the interhemispheric contrast, analogous to the discriminative signal) exhibits a highly robust and significant negative correlation with RT across all contextual variations and throughout all three timepoints. In strict contrast, PC_Shared (analogous to the global cognitive readiness signal) and the minor spatial components (PC_Hand, PC_Hemi) demonstrate weak and inconsistent associations with RT. Values within cells denote Pearson's r and their corresponding P values.

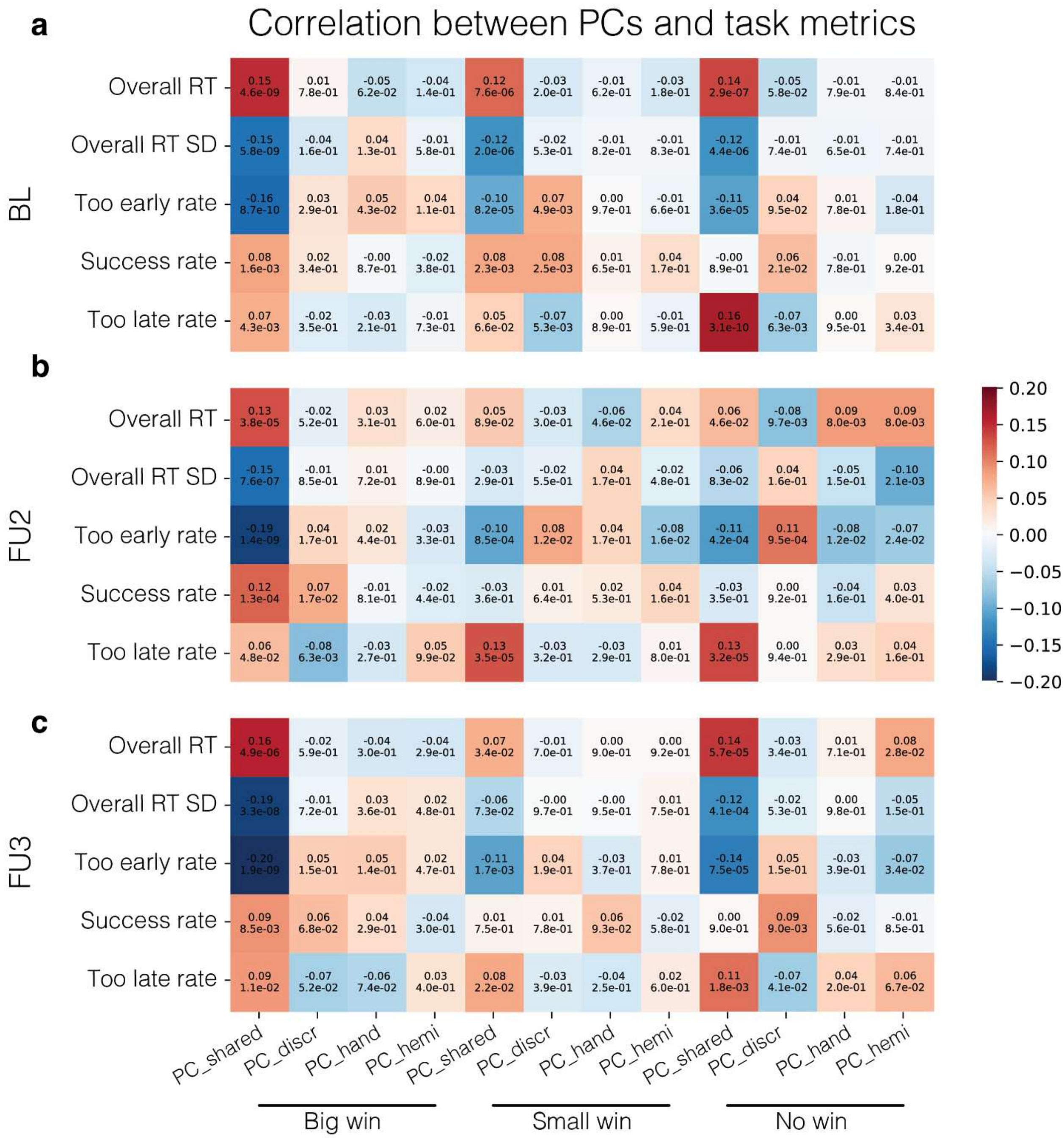

Fig. S6. Associations between model-free principal components and global task performance across longitudinal timepoints. (**a** to **c**) Heatmaps displaying the Pearson correlation coefficients (r, top values in each cell) and corresponding P values (bottom values) between the four principal components (PCs) extracted via model-free PCA (PC_Shared, PC_Discr, PC_Hand, PC_Hemi) and five global task performance metrics (Overall RT, Overall RT SD, Too Early Rate, Success

Rate, Too Late Rate). The correlation matrices are systematically stratified by longitudinal timepoints: Baseline (**a**), Follow-up 2 (**b**), and Follow-up 3 (**c**), and further divided by the task reward conditions (Big Win, Small Win, No Win) from which the PCs were derived. Complementing the execution speed findings in Fig. S5, these matrices robustly confirm the second half of the double dissociation framework. PC_Shared (analogous to the global cognitive readiness signal) exhibits highly consistent associations with global temporal precision and vigilance across all timepoints and contextual variations. Stronger PC_Shared activation reliably predicts a more concentrated and precise response pattern (e.g., lower Overall RT SD, lower Too Early Rate) and generally correlates with a higher Success Rate. In contrast, PC_Discr, which powerfully predicted success-trial RT (Fig. S5), shows virtually no association with these global performance metrics. Minor spatial components (PC_Hand, PC_Hemi) similarly lack consistent behavioural relevance. Values within cells denote Pearson's r and their corresponding P values.

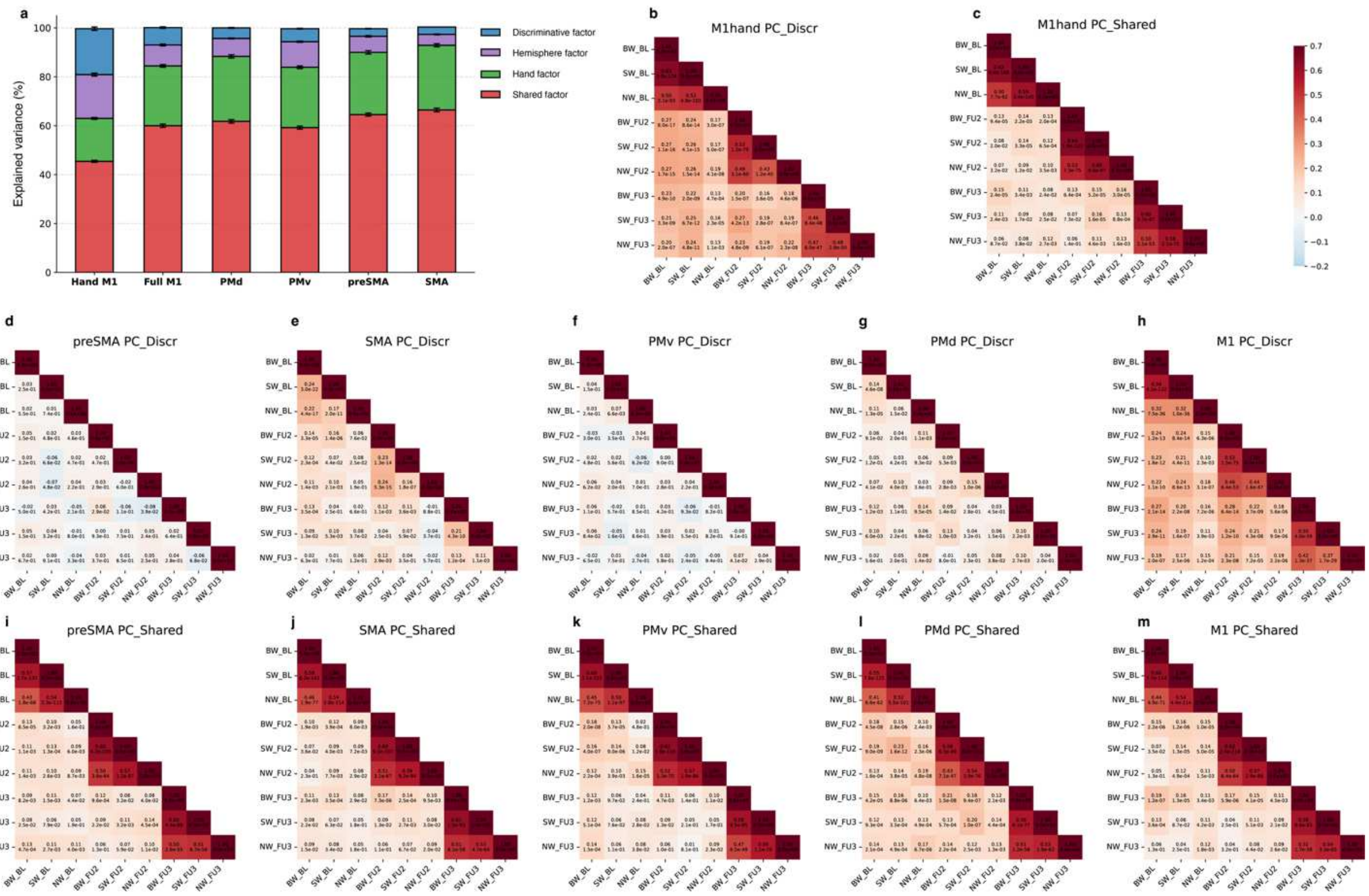

Fig. S7. Variance explanation and longitudinal stability of model-free latent components across extended cortical motor regions. (**a**) Bar chart comparing the percentage of total variance explained by the four principal components across six predefined cortical motor regions (Hand M1, Full M1, PMd, PMv, preSMA, SMA). Error bars indicate the standard error of the mean across all task conditions and timepoints. While the shared component dominates the explained variance across all evaluated motor regions, the discriminative component accounts for a uniquely high proportion of variance specifically within the localised Hand M1 region (~20%). In contrast, the discriminative variance drops significantly (<8.1%) in all other extended motor areas. (**b** to **m**) Correlation matrices assessing the temporal and contextual stability of the discriminative (**b**, **d** to **h**) and shared (**c**, **i** to **m**) components extracted from the extended motor ROIs. Values within cells denote Pearson's r and their corresponding P values.

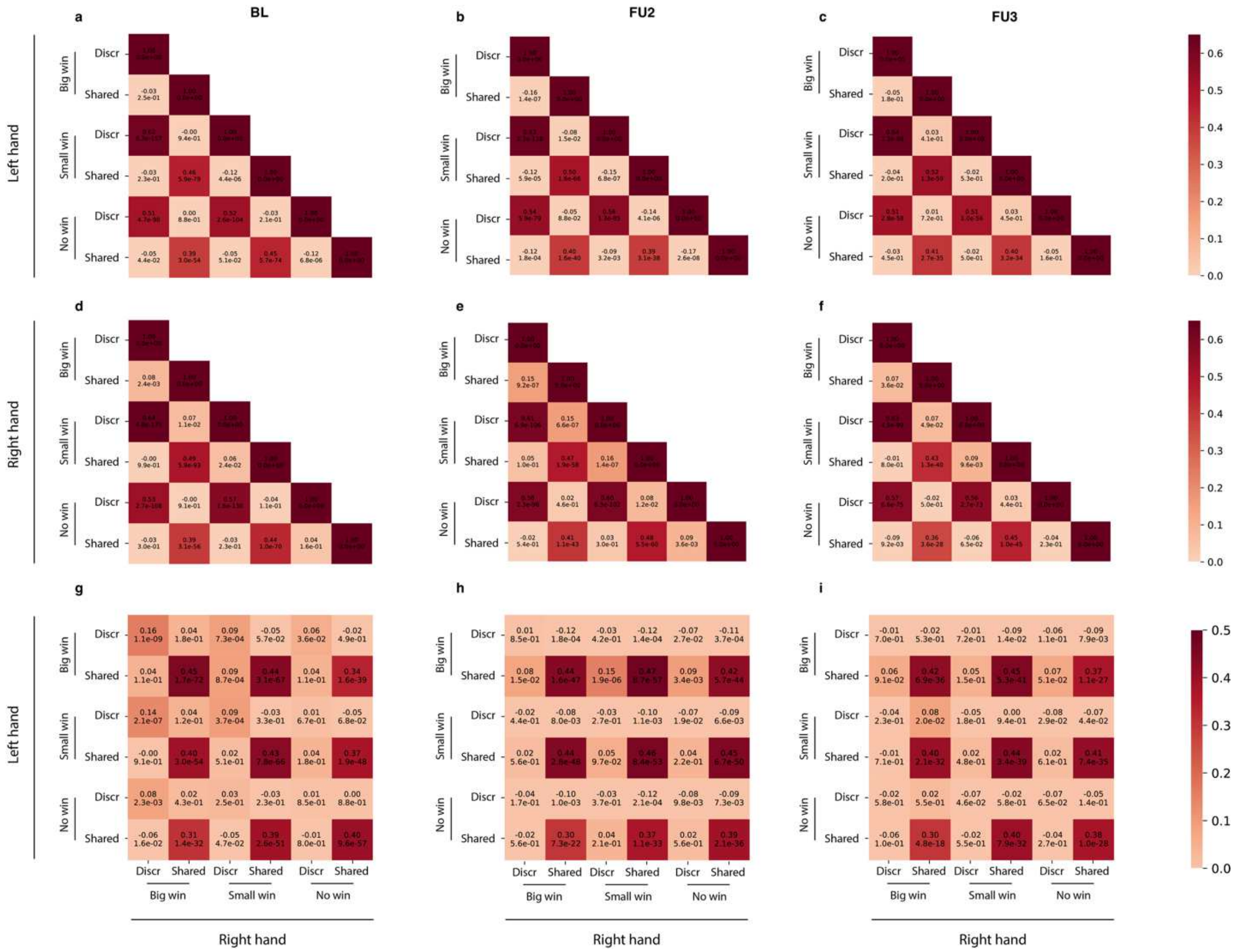

Fig. S8. Longitudinal stability and orthogonality of the hand-specific decomposed motor latent signals. (**a** to **i**) Heatmaps illustrating the intra- and inter-class Pearson correlation coefficients between the discriminative (Discr) and shared latent signals derived from single-hand responses across varying reward contexts (Big Win, Small Win, No Win). (**a** to **c**) Correlation matrices within left-hand responses at Baseline (BL, **a**), Follow-up 2 (FU2, **b**), and Follow-up 3 (FU3, **c**). (**d** to **f**) Correlation matrices within right-hand responses at BL (**d**), FU2 (**e**), and FU3 (**f**). The robust intra-class correlations (Shared-Shared and Discr-Discr) and the weak inter-class correlations validate the functional orthogonality of the signal decomposition model. (**g** to **i**) Cross-hand correlation matrices between left- and right-hand decomposed signals at BL (**g**), FU2 (**h**), and FU3 (**i**). The cross-hand comparisons demonstrate that while the shared latent signals,

representing global cognitive readiness, remain strongly correlated across hands, the discriminative latent signals, representing lateralised kinematic preparation, are largely uncorrelated, confirming their hand-specific kinematic regulatory roles. Values within cells denote Pearson's r and their corresponding P values.

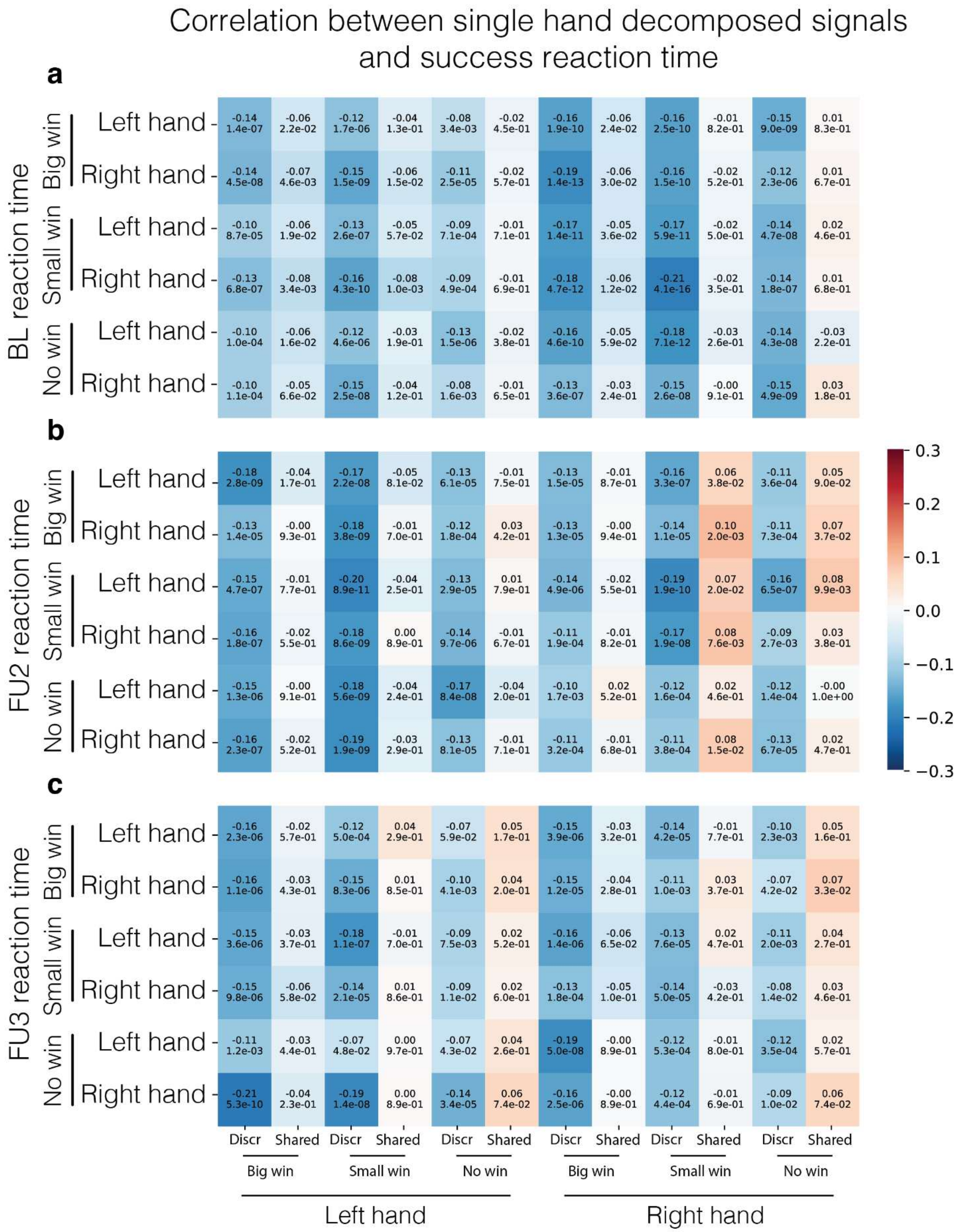

Fig. S9. Hand-specific discriminative latent signals selectively predict execution speed during

successful motor responses. (**a** to **c**) Correlation matrices mapping the relationship between single-hand decomposed latent signals (discriminative and shared) and reaction times (RT) in successful trials across reward conditions (Big Win, Small Win, No Win) at Baseline (BL, **a**), Follow-up 2 (FU2, **b**), and Follow-up 3 (FU3, **c**). The color scale represents the Pearson correlation coefficient (r), with corresponding P values annotated within each cell. The hand-specific discriminative latent signal consistently exhibits robust negative correlations with RT, indicating that higher discriminative activation specifically facilitates faster execution speeds. In contrast, the bi-hemispheric shared latent signal exhibits weak or non-significant associations with execution speed, reinforcing the double dissociation between the kinematic execution and the cognitive readiness network. Values within cells denote Pearson's r and their corresponding P values.

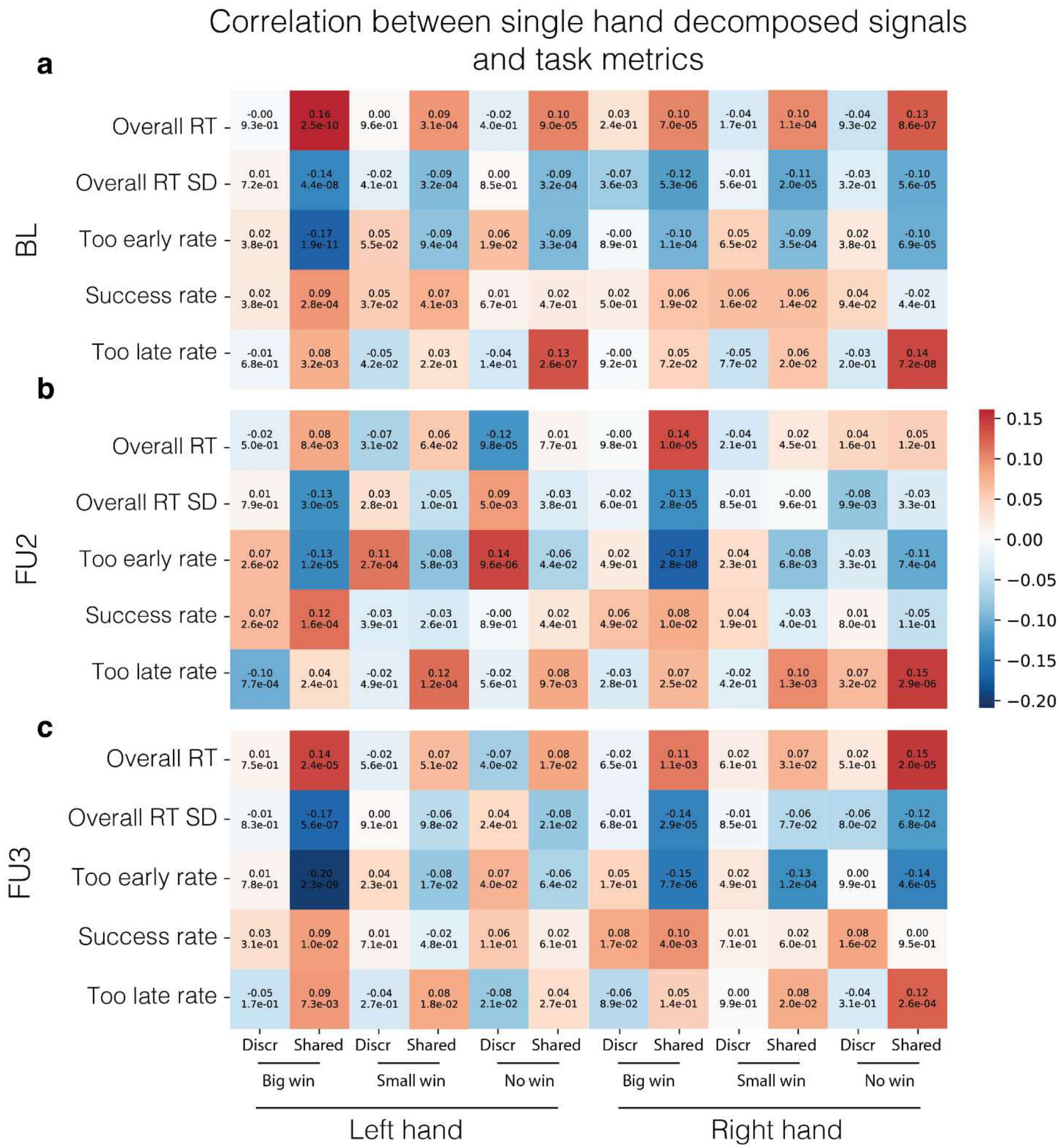

Fig. S10. Hand-specific shared latent signals globally predict temporal precision and overall task performance. (**a** to **c**) Heatmaps detailing the correlations between decomposed single-hand latent signals and global task performance metrics (Overall RT, Overall RT SD, Too Early Rate, Success Rate, Too Late Rate). Analyses were conducted across the three longitudinal timepoints: Baseline (BL, **a**), Follow-up 2 (FU2, **b**), and Follow-up 3 (FU3, **c**). Unlike the discriminative

signal, the shared latent signal shows robust, longitudinally stable associations with global metrics of temporal precision. Specifically, stronger shared signals correlate with reduced RT variability (Overall RT SD) and lower premature response rates (Too Early Rate) across varying incentive contexts. These distinct behavioural signatures underscore the shared component's cognitive readiness role in motor initiation. Values within cells denote Pearson's r and their corresponding P values.

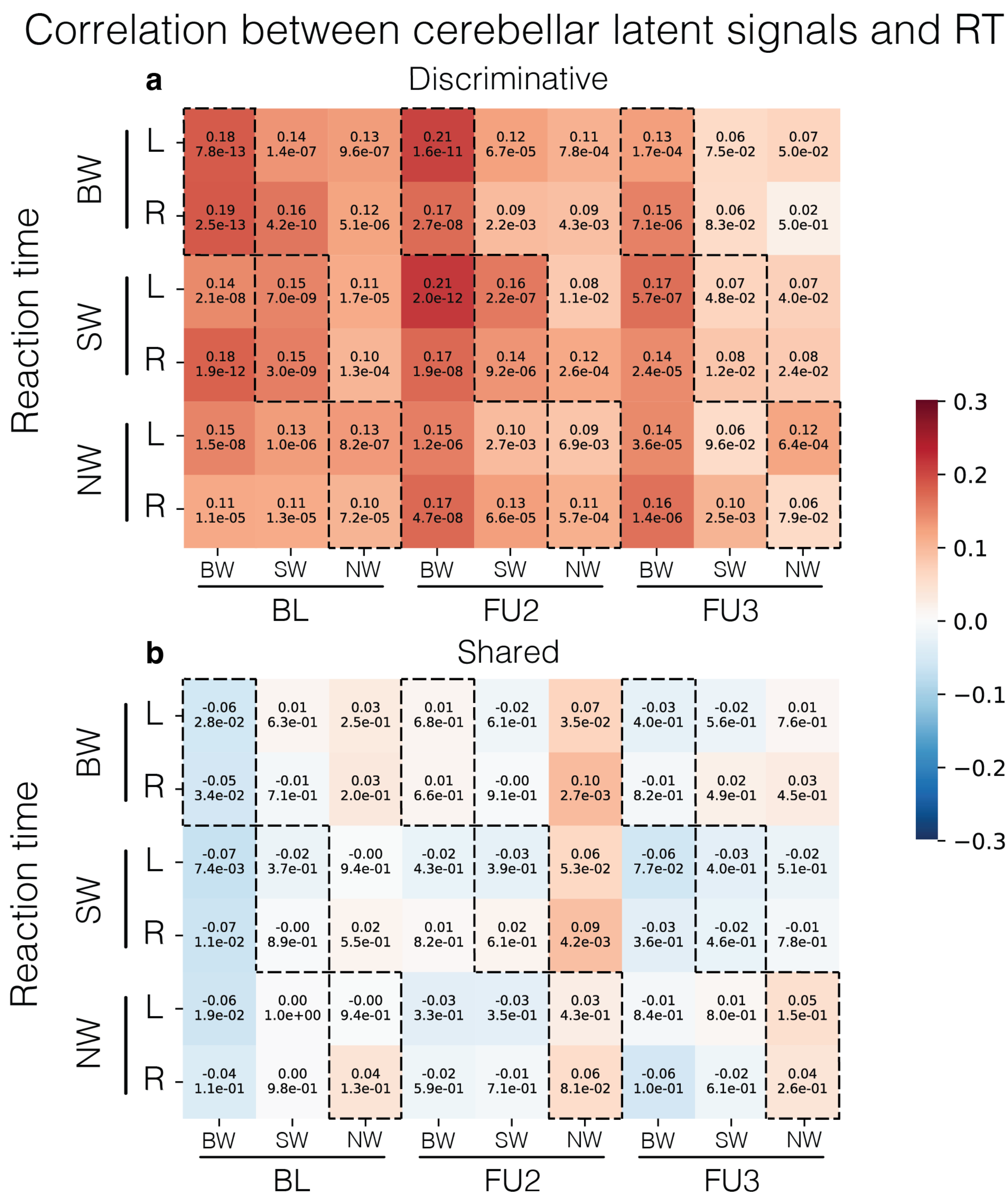

Fig. S11. Dissociable correlations between decomposed cerebellar latent signals and condition-specific reaction times. Heatmaps detailing the Pearson correlation coefficients between the decomposed cerebellar latent signals, the hand-specific discriminative component (**a**)

and the bi-hemispheric shared component (**b**), and success-trial reaction times (RT). Correlations are mapped across multiple reward valences (BW: Big Win; SW: Small Win; NW: No Win) and corresponding response hands (L: left; R: right) over three longitudinal timepoints (BL, FU2, FU3). The discriminative latent signal (a) demonstrates robust associations with RT, reinforcing its localised role in kinematic preparation. In contrast, the shared latent signal (b) lacks specific correlations with individual trial RTs. Values within cells denote Pearson's r and their corresponding P values.

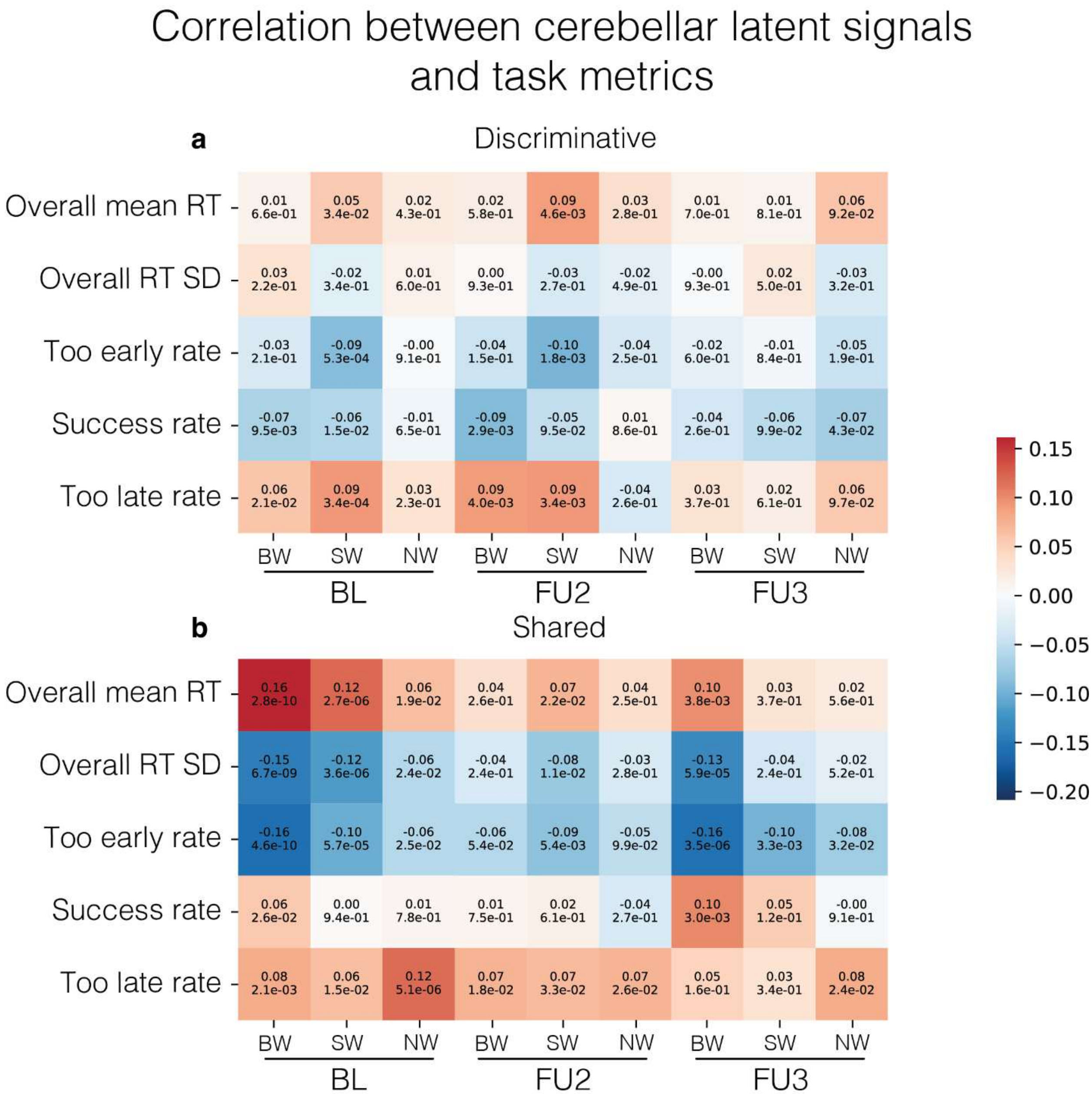

Fig. S12. Dissociable correlations between decomposed cerebellar latent signals and global task performance. Correlation matrices illustrating the relationship between cerebellar latent signals, discriminative (**a**) and shared (**b**), and global task performance indices. Consistent with a cognitive readiness framework, the shared latent component (b) is robustly associated with indices of response precision, specifically driving decreased RT variability (Overall RT SD) and a reduced rate of premature responses (Too Early Rate), most prominently under high-reward conditions

(BW). Conversely, the discriminative kinematic component (a) exhibits weak associations with these global performance and temporal vigilance measures. Values within cells represent Pearson's r and associated P values.

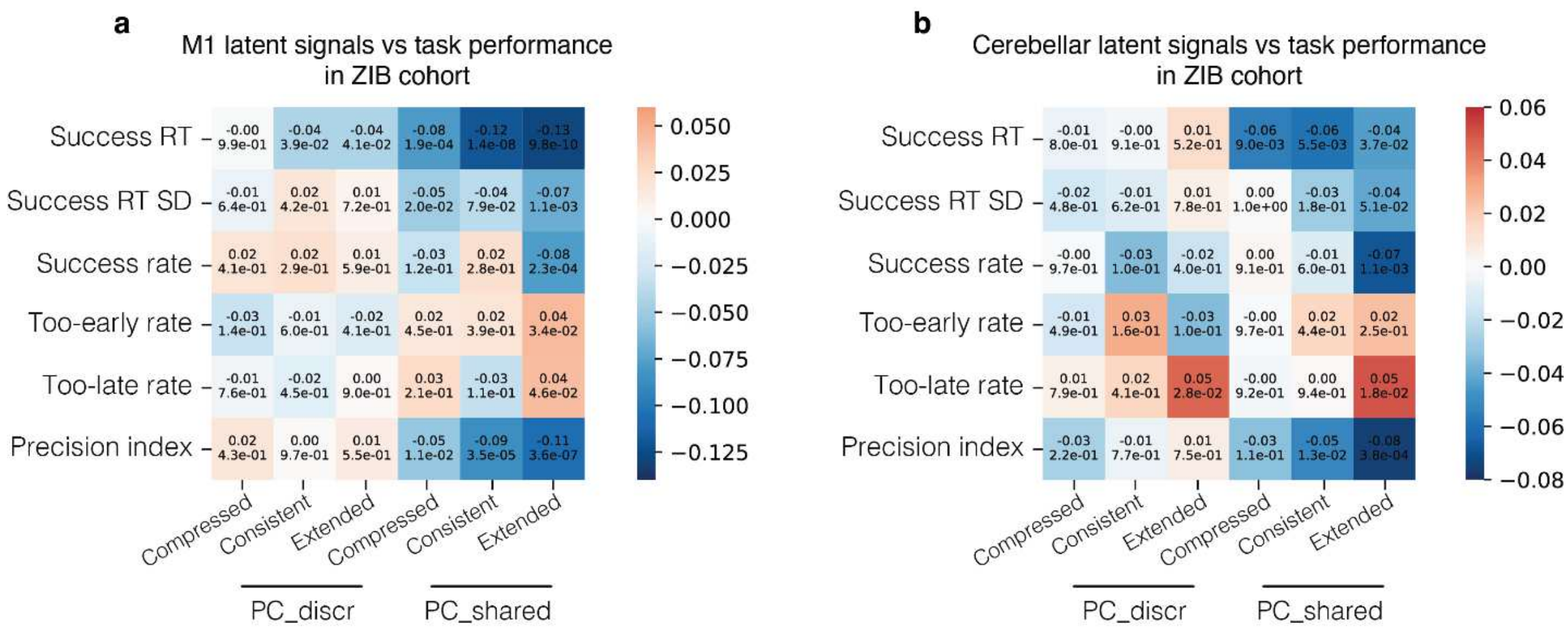

Fig. S13. Generalisability of the cognitive-motor dual regulation model across distinct temporal contexts in the ZIB cohort. Validation of the dual-system architecture in the independent ZIB cohort using model-free principal component analysis for both the primary motor cortex (M1, **a**) and cerebellum (**b**). Trials are stratified by temporal expectancy contexts based on inter-trial delay variations: compressed (Δ<0 s), consistent ($0 \leq \Delta \leq 1$ s), and extended ($\Delta > 1$ s). Values within cells represent Pearson's r and associated P values.

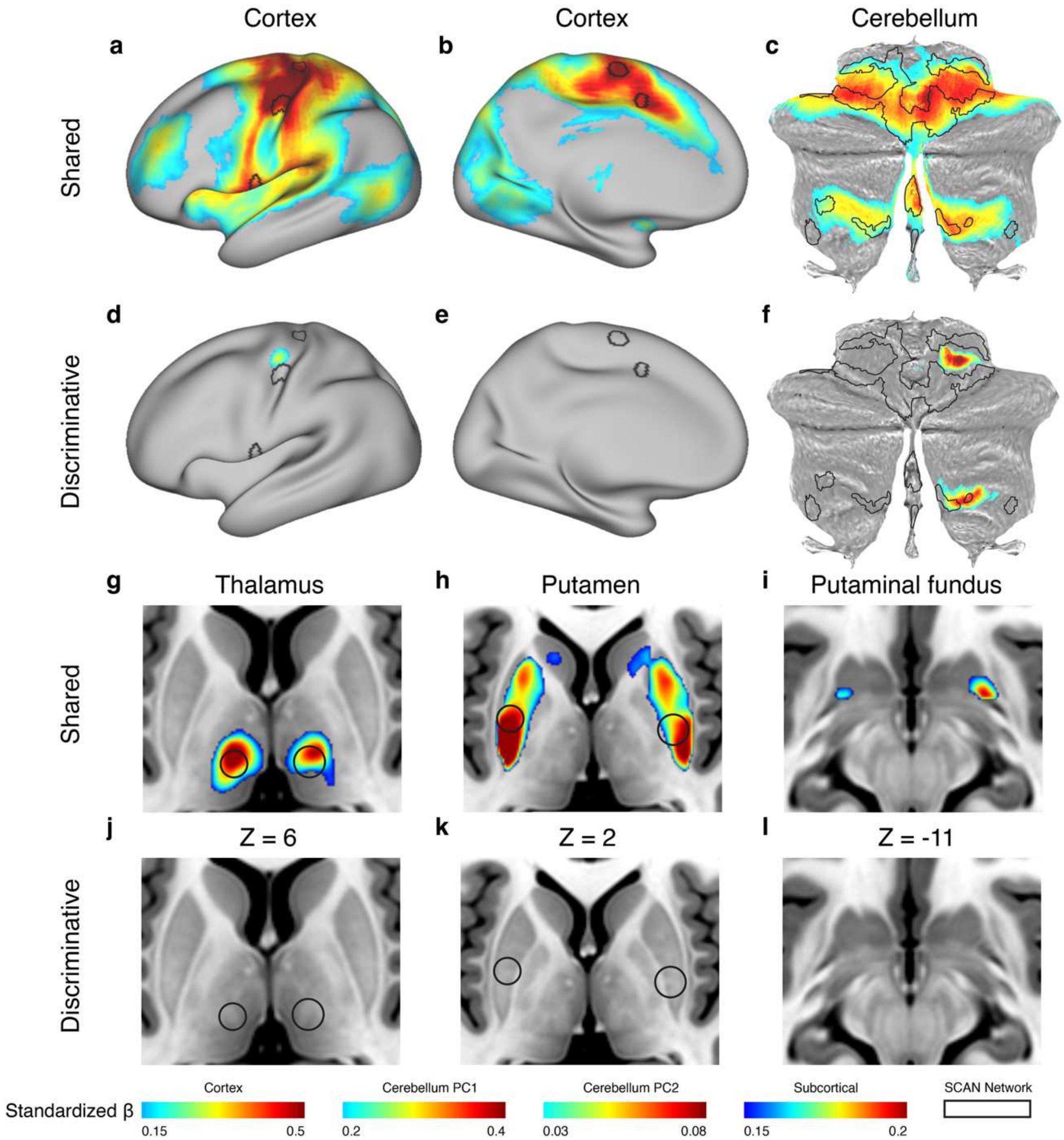

Fig. S14. Brain-wide anatomical dissociation of cognitive readiness and kinematic preparation networks in compressed trials of the ZIB cohort. (**a** to **c**) Brain-wide contribution maps of the hand-invariant shared signal (cognitive readiness) across the cerebral cortex (**a** and **b**) and cerebellar flatmap (**c**). The shared signal exhibits broad, bilateral recruitment. Black outlines denote the a priori defined Somato-Cognitive Action Network (SCAN) regions. Notably, the spatial distribution of the shared signal in M1 shows a striking concordance with the inter-effector,

cognitive-only SCAN nodes. (**d** to **f**) Brain-wide contribution maps of the cross-hemispheric discriminative signal (kinematic preparation). In strict contrast to the shared signal, the discriminative signal is highly localised to the classical effector-specific motor territories (e.g., the M1 hand-knob) and spares the SCAN regions. (**g** to **l**) Subcortical contribution mapping reveals a profound structural dissociation. The shared signal (**g** to **i**) robustly recruits the central cortico-striato-thalamic loop, with prominent bilateral engagement of the thalamus (**g**) and the putamen (**h**), and especially, the putaminal fundus (**i**). Conversely, the discriminative signal (**j** to **l**) exhibits minimal to no engagement of these subcortical areas. For visualisation purposes, statistical maps in the subcortical panels are masked to the anatomical boundaries of the thalamus and putamen displayed.

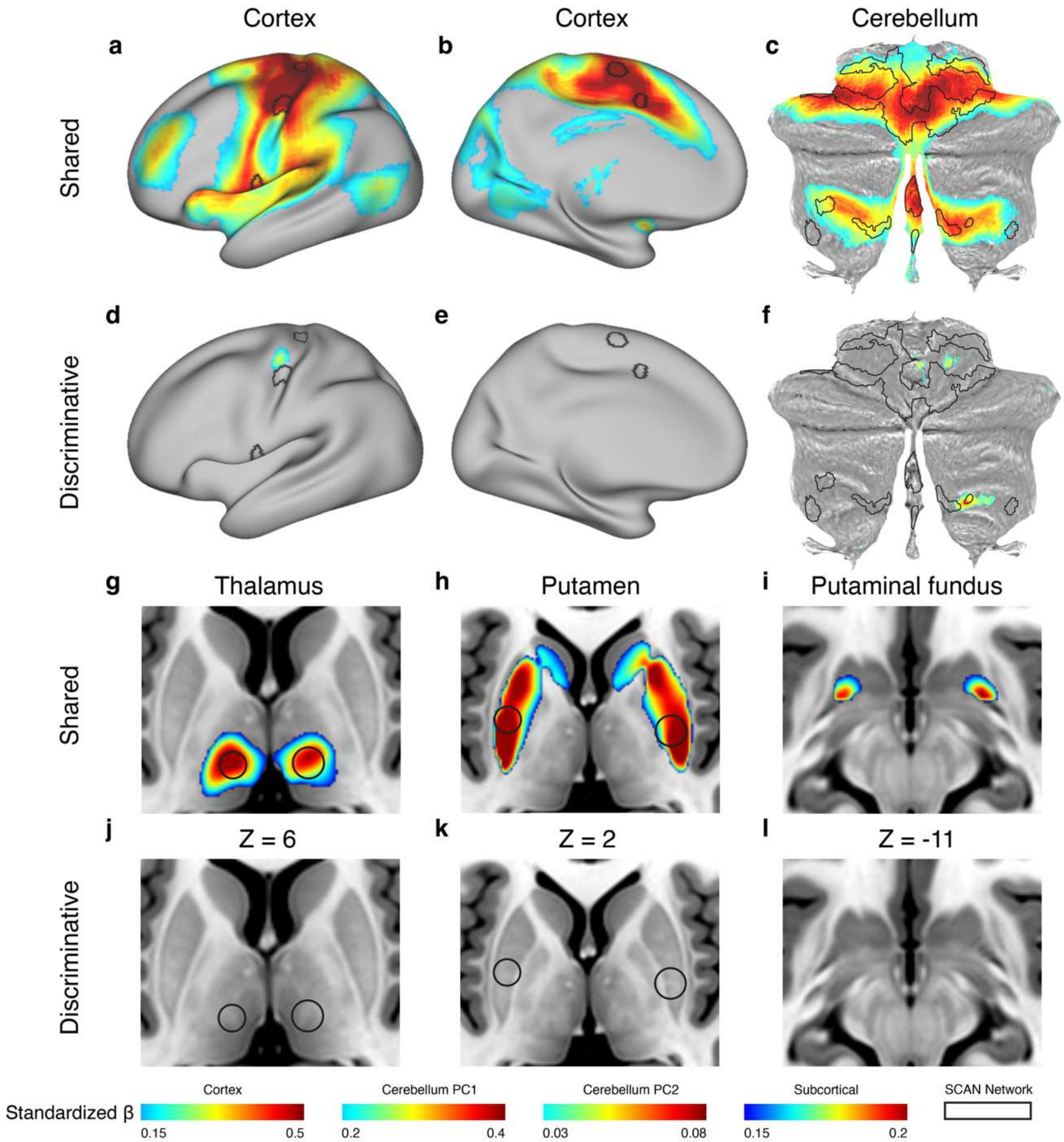

Fig. S15. Brain-wide anatomical dissociation of cognitive readiness and kinematic preparation networks in extended trials of the ZIB cohort. (**a** to **c**) Brain-wide contribution maps of the hand-invariant shared signal (cognitive readiness) across the cerebral cortex (**a** and **b**) and cerebellar flatmap (**c**). The shared signal exhibits broad, bilateral recruitment. Black outlines denote the a priori defined Somato-Cognitive Action Network (SCAN) regions. Notably, the spatial distribution of the shared signal in M1 shows a striking concordance with the inter-effector,

cognitive-only SCAN nodes. (**d** to **f**) Brain-wide contribution maps of the cross-hemispheric discriminative signal (kinematic preparation). In strict contrast to the shared signal, the discriminative signal is highly localised to the classical effector-specific motor territories (e.g., the M1 hand-knob) and spares the SCAN regions. (**g** to **l**) Subcortical contribution mapping reveals a profound structural dissociation. The shared signal (**g** to **i**) robustly recruits the central cortico-striato-thalamic loop, with prominent bilateral engagement of the thalamus (**g**) and the putamen (**h**), and especially, the putaminal fundus (**i**). Conversely, the discriminative signal (**j** to **l**) exhibits minimal to no engagement of these subcortical areas. For visualisation purposes, statistical maps in the subcortical panels are masked to the anatomical boundaries of the thalamus and putamen displayed.

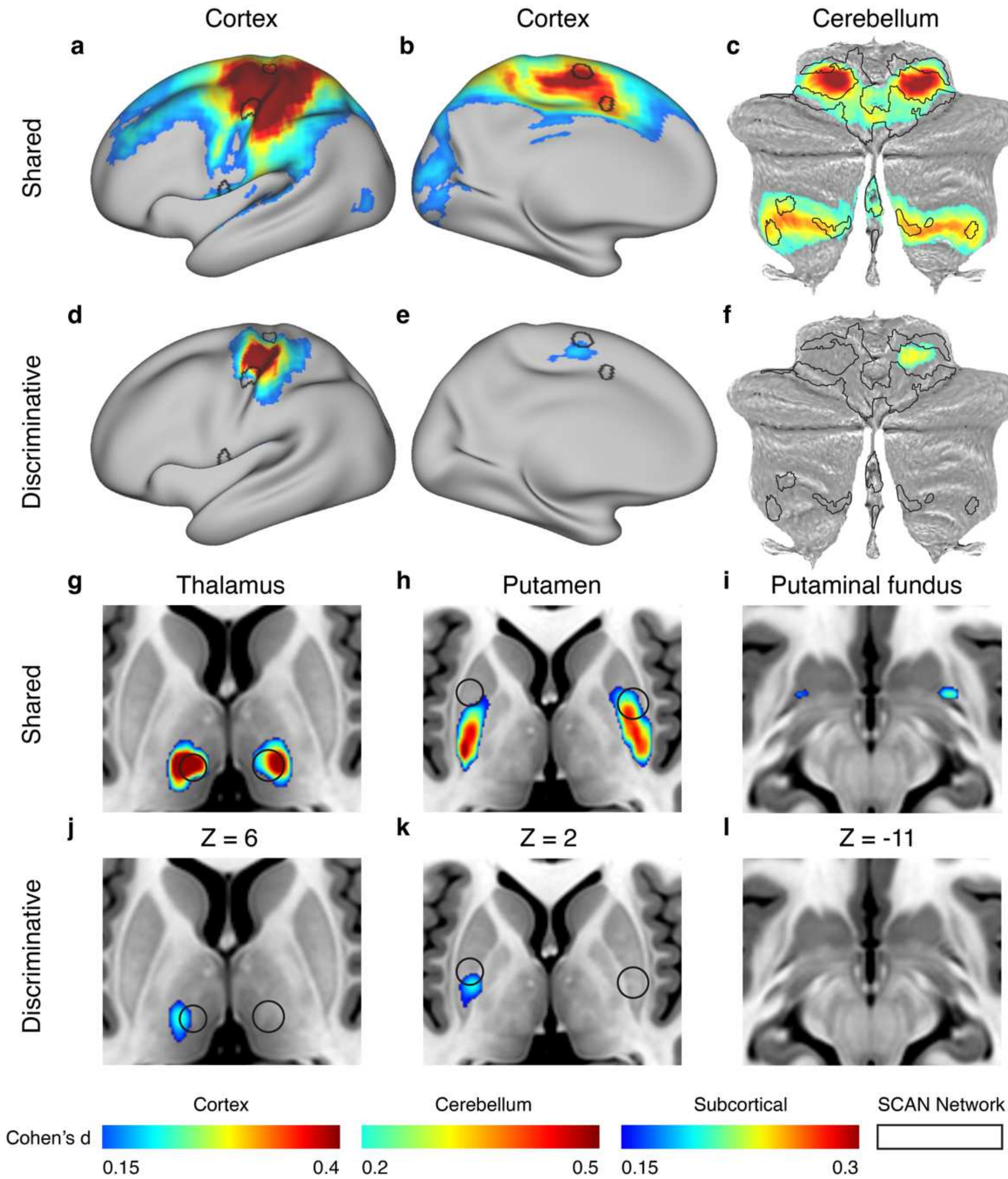

Fig. S16. Task-based functional connectivity corroborates the brain-wide anatomical dissociation of cognitive readiness and kinematic preparation networks. (**a** to **l**)Brain-wide seed-to-voxel functional connectivity (FC) maps derived from the decomposed latent signals during the anticipation phase of successful trials (Baseline, Big Win as an example). Consistent with the task-activation maps, the hand-invariant shared FC signal (cognitive readiness) exhibits broad, bilateral functional synchronisation with inter-effector SCAN regions and key subcortical hubs. In contrast, the cross-hemispheric discriminative FC signal (kinematic preparation)

demonstrates highly localised, lateralised connectivity, predominantly restricted to the classical effector-specific motor territories, while actively sparing the SCAN regions. For visualisation purposes, statistical maps in the subcortical panels are masked to the anatomical boundaries of the thalamus and putamen displayed.

Supplementary Table 1. Peak activations within the bilateral M1 hand knob area during reward anticipation. Summary of peak MNI coordinates, statistical significance, and effect sizes (Cohen's d) for the contrast of big-win versus no-win during the anticipation phase of the MID task. Significant positive activations were consistently observed in both the contralateral and ipsilateral hand knob areas of the primary motor cortex across all three timepoints (Baseline, Follow-up 2, and Follow-up 3) during both left- and right-hand responses.

Timepoint	Responding Hand	Hemisphere (M1)	Peak MNI (x, y, z)	Peak t-value	Cohen's d	p-value
Baseline	Right	Contralateral (Left)	-39, -24, 57	21.66	0.55	5.36E-91
		Ipsilateral (Right)	42, -12, 51	11.35	0.29	9.99E-29
	Left	Contralateral (Right)	39, -21, 60	21.08	0.54	7.70E-87
		Ipsilateral (Left)	-39, -18, 57	9.02	0.23	5.47E-19
Follow-up 2	Right	Contralateral (Left)	-42, -21, 57	13.90	0.40	2.00E-36
		Ipsilateral (Right)	42, -12, 54	8.72	0.27	1.05E-17
	Left	Contralateral (Right)	42, -12, 51	12.79	0.39	5.87E-35
		Ipsilateral (Left)	-39, -18, 57	4.46	0.14	9.06E-06
Follow-up 3	Right	Contralateral (Left)	-42, -21, 57	11.64	0.39	2.88E-29
		Ipsilateral (Right)	42, -12, 51	4.95	0.16	8.86E-07
	Left	Contralateral (Right)	36, -24, 57	11.99	0.40	7.82E-31
		Ipsilateral (Left)	-39, -18, 57	5.62	0.19	2.55E-08

Supplementary Table 2. Quantitative evaluation of bilateral M1 activation and inter-hemispheric differences. Summary of mean activations, statistical significance, and effect sizes for the functionally defined hand-knob ROIs in the primary motor cortex (M1) across all timepoints, reward conditions, and responding hands. Paired t-tests reveal that while both hemispheres are significantly engaged, contralateral activations are drastically stronger than their ipsilateral counterparts across all contexts.

Timepoint	Condition	Responding Hand	Contra M1	Contra Mean (SD)	Contra t	Contra d	Contra *P*	Ipsi M1	Ipsi Mean (SD)	Ipsi t	Ipsi d	Ipsi *P*	Paired t (Contra -Ipsi)	Paired dz	Paired *P*
Baseline	Big-win	Right	Left	1.000 (1.165)	33.64	0.86	2.47E-186	Right	-0.684 (1.074)	-24.97	-0.64	8.58E-116	48.65	1.24	2.881E-313
		Left	Right	1.001 (1.144)	34.28	0.87	9.50E-192	Left	-0.741 (1.167)	-24.88	-0.63	4.12E-115	49.21	1.26	6.623E-318
	Small-win	Right	Left	0.522 (1.135)	18.05	0.46	3.60E-66	Right	-0.871 (1.077)	-31.72	-0.81	2.94E-170	41.24	1.05	8.06E-251
		Left	Right	0.603 (1.024)	23.08	0.59	1.87E-101	Left	-0.833 (1.153)	-28.34	-0.72	1.92E-142	43.98	1.12	4.61E-274
	No-win	Right	Left	0.249 (1.136)	8.60	0.22	1.99E-17	Right	-0.956 (1.102)	-34.00	-0.87	2.28E-189	37.17	0.95	3.16E-216
		Left	Right	0.311 (1.006)	12.11	0.31	2.60E-32	Left	-0.945 (1.172)	-31.61	-0.81	2.63E-169	39.46	1.01	1.04E-235
Follow-up 2	Big-win	Right	Left	1.073 (1.319)	26.62	0.81	3.12E-120	Right	-0.531 (1.111)	-15.65	-0.48	6.91E-50	34.79	1.06	5.34E-178
		Left	Right	1.050 (1.169)	29.41	0.90	7.60E-140	Left	-0.541 (1.282)	-13.81	-0.42	4.73E-40	34.98	1.07	2.22E-179
	Small-win	Right	Left	0.614 (1.350)	14.87	0.45	1.25E-45	Right	-0.646 (1.119)	-18.89	-0.58	6.16E-69	26.89	0.82	4.09E-122
		Left	Right	0.538 (1.111)	15.84	0.48	6.09E-51	Left	-0.626 (1.237)	-16.56	-0.51	5.00E-55	26.57	0.81	7.31E-120
	No-win	Right	Left	0.448 (1.193)	12.29	0.38	1.39E-32	Right	-0.686 (1.070)	-20.98	-0.64	3.81E-82	26.88	0.82	5.17E-122
		Left	Right	0.474 (1.223)	12.67	0.39	2.23E-34	Left	-0.703 (1.413)	-16.27	-0.50	2.43E-53	25.10	0.77	1.16E-109
Follow-up 3	Big-win	Right	Left	1.017 (1.285)	23.73	0.79	4.94E-97	Right	-0.583 (1.165)	-15.02	-0.50	1.14E-45	32.30	1.08	1.55E-152
		Left	Right	1.053 (1.212)	26.05	0.87	6.44E-112	Left	-0.535 (1.293)	-12.40	-0.41	9.99E-33	32.30	1.08	1.56E-152
	Small-win	Right	Left	0.642 (1.273)	15.14	0.50	2.87E-46	Right	-0.659 (1.201)	-16.46	-0.55	2.09E-53	25.73	0.86	7.41E-110
		Left	Right	0.582 (1.102)	15.86	0.53	4.12E-50	Left	-0.607 (1.177)	-15.48	-0.52	4.51E-48	25.64	0.85	2.89E-109
	No-win	Right	Left	0.463 (1.121)	12.39	0.41	1.15E-32	Right	-0.656 (1.148)	-17.15	-0.57	2.86E-57	25.18	0.84	2.69E-106
		Left	Right	0.495 (1.214)	12.24	0.41	5.85E-32	Left	-0.736 (1.300)	-16.99	-0.57	2.51E-56	24.98	0.83	4.76E-105

Supplementary Table 3. Summary of linear mixed-effects models (LMMs) predicting reaction times from bilateral M1 activations. The table presents the standardized coefficients β, 95% confidence intervals (CI), and p-values for the primary predictors of interest (Contralateral and Ipsilateral M1 signals) across single-hemisphere and dual-hemisphere LMMs. Controlling for the opposite hemisphere in the Dual models mutually strengthens the lateralized behavioral associations.

Dependent Variable	Model Type	Predictor	Estimate (β)	95% CI	z-value	p-value
Left-Hand RT	Single (Contra)	Contralateral (rM1)	-0.084	[-0.102, -0.067]	-9.322	1.14E-20
	Single (Ipsi)	Ipsilateral (lM1)	0.032	[0.015, 0.049]	3.616	3.00E-04
	Dual (Both)	Contralateral (rM1)	-0.104	[-0.123, -0.086]	-11.003	3.70E-28
		Ipsilateral (lM1)	0.063	[0.045, 0.081]	6.854	7.20E-12
Right-Hand RT	Single (Contra)	Contralateral (lM1)	-0.058	[-0.076, -0.040]	-6.416	1.40E-10
	Single (Ipsi)	Ipsilateral (rM1)	0.06	[0.043, 0.078]	6.711	1.93E-11
	Dual (Both)	Contralateral (lM1)	-0.083	[-0.102, -0.065]	-8.81	1.25E-18
		Ipsilateral (rM1)	0.084	[0.066, 0.103]	9.029	1.73E-19

Supplementary Table 4. Enhanced prediction of reaction time by integrating ipsilateral M1 activation in multivariate linear regression models. Summary of model comparison statistics evaluating the predictive performance for reaction time (RT) across all longitudinal timepoints, task conditions, and responding hands. Model 1 utilizes contralateral M1 activation alone as the primary predictor, whereas Model 2 incorporates both contralateral and ipsilateral M1 activations. The table reports the adjusted R^2 for both models, alongside the estimate (β), standard error (SE), and statistical significance (t and P values) of the newly introduced ipsilateral activation term. The Model Comparison F-statistic and its associated P-value confirm that the inclusion of ipsilateral M1 signals consistently and significantly improves the explanatory power for RT beyond what is achieved by the contralateral M1 alone, underscoring the independent functional contribution of the ipsilateral hemisphere to motor execution speed.

Timepoint	Task Condition	Hand Used	Model 1 Adj. R^2	Model 2 Adj. R^2	Ipsi. Activation β (SE)	Ipsi. Activation t	Ipsi. Activation P	Model Comparison F	df1 (Numerator)	df2 (Denominator)	Model Comparison *P*
BL	Big Win	Right	0.096	0.107	2.960 (0.666)	4.44	9.48E-06	19.75	1	1505	9.48E-06
		Left	0.093	0.098	1.744 (0.606)	2.88	4.06E-03	8.28	1	1513	4.06E-03
	No Win	Right	0.055	0.075	4.377 (0.775)	5.65	1.97E-08	31.88	1	1451	1.97E-08
		Left	0.079	0.086	2.474 (0.733)	3.37	7.60E-04	11.39	1	1437	7.60E-04
	Small Win	Right	0.075	0.095	4.015 (0.683)	5.88	5.12E-09	34.54	1	1505	5.12E-09
		Left	0.081	0.086	2.036 (0.656)	3.10	1.96E-03	9.62	1	1507	1.96E-03
FU2	Big Win	Right	0.217	0.224	2.464 (0.745)	3.31	9.77E-04	10.93	1	1054	9.77E-04
		Left	0.233	0.243	2.237 (0.580)	3.86	1.20E-04	14.90	1	1061	1.20E-04
	No Win	Right	0.196	0.205	2.929 (0.833)	3.51	4.61E-04	12.35	1	1000	4.61E-04
		Left	0.193	0.204	2.544 (0.665)	3.83	1.37E-04	14.66	1	989	1.37E-04
	Small Win	Right	0.187	0.209	4.225 (0.764)	5.53	4.09E-08	30.55	1	1055	4.09E-08
		Left	0.230	0.249	3.653 (0.690)	5.29	1.45E-07	28.03	1	1060	1.45E-07
FU3	Big Win	Right	0.258	0.264	2.166 (0.791)	2.74	6.28E-03	7.50	1	890	6.28E-03
		Left	0.236	0.246	2.596 (0.750)	3.46	5.63E-04	11.98	1	874	5.63E-04
	No Win	Right	0.187	0.196	3.137 (0.938)	3.35	8.58E-04	11.19	1	853	8.58E-04
		Left	0.203	0.211	2.548 (0.811)	3.14	1.75E-03	9.86	1	830	1.75E-03
	Small Win	Right	0.276	0.278	1.641 (0.818)	2.01	4.52E-02	4.02	1	893	4.52E-02
		Left	0.256	0.268	3.401 (0.851)	4.00	6.95E-05	15.98	1	884	6.95E-05

Supplementary Table 5. Longitudinal stability of decomposed latent signals versus standard M1 activations. Summary of cross-timepoint correlations comparing the temporal reliability of the undecomposed contralateral M1 BOLD signal (Activation r) against the orthogonally decomposed components (Latent Signal r). Dunn and Clark's Z tests were employed to statistically compare these dependent correlation coefficients, utilizing the corresponding cross-correlations (Cross r13, r1, r23, r24) among the variables. Across task conditions and responding hands, the discriminative signal consistently demonstrates significantly higher longitudinal reliability (greater r values and significant Z scores) across Baseline (BL), Follow-up 2 (FU2), and Follow-up 3 (FU3), indicative of a stable, trait-like functional architecture. Conversely, the shared signal exhibits longitudinal stability comparable to standard undecomposed activations (non-significant Z scores), reflecting state-dependent arousal fluctuations.

Task Condition	Signal Type	Comparison	Hand Used	Activation r	Latent Signal r	Cross r13	Cross r14	Cross r23	Cross r24	Z	P Value
Big Win	Discriminative	BL vs. FU2	Left	0.164	0.298	0.454	0.103	0.208	0.443	3.46	2.74E-04
		BL vs. FU2	Right	0.145	0.298	0.507	0.146	0.134	0.427	3.96	3.82E-05
		BL vs. FU3	Left	0.183	0.264	0.469	0.129	0.091	0.413	1.86	3.16E-02
		BL vs. FU3	Right	0.162	0.248	0.516	0.163	0.114	0.447	1.99	2.32E-02
		FU2 vs. FU3	Left	0.156	0.282	0.423	0.072	0.154	0.396	2.78	2.70E-03
		FU2 vs. FU3	Right	0.094	0.279	0.424	0.119	0.106	0.433	4.08	2.27E-05
	Shared	BL vs. FU2	Left	0.161	0.146	0.690	0.134	0.100	0.632	-0.40	6.56E-01
		BL vs. FU2	Right	0.150	0.156	0.682	0.121	0.109	0.707	0.18	4.30E-01
		BL vs. FU3	Left	0.167	0.183	0.679	0.128	0.162	0.660	0.41	3.41E-01
		BL vs. FU3	Right	0.172	0.197	0.707	0.115	0.147	0.680	0.63	2.64E-01
		FU2 vs. FU3	Left	0.156	0.149	0.635	0.123	0.121	0.646	-0.17	5.66E-01
		FU2 vs. FU3	Right	0.088	0.157	0.710	0.101	0.063	0.682	1.65	4.99E-02
Small Win	Discriminative	BL vs. FU2	Left	0.074	0.280	0.382	0.103	0.155	0.397	5.09	1.83E-07
		BL vs. FU2	Right	0.133	0.271	0.428	0.107	0.156	0.448	3.52	2.17E-04
		BL vs. FU3	Left	0.109	0.254	0.404	0.130	0.087	0.394	3.27	5.37E-04
		BL vs. FU3	Right	0.123	0.240	0.442	0.110	0.106	0.448	2.68	3.64E-03
		FU2 vs. FU3	Left	0.120	0.260	0.413	0.107	0.124	0.378	3.04	1.16E-03
		FU2 vs. FU3	Right	0.113	0.267	0.445	0.082	0.109	0.419	3.41	3.28E-04
	Shared	BL vs. FU2	Left	0.087	0.139	0.666	0.124	0.047	0.631	1.36	8.68E-02
		BL vs. FU2	Right	0.139	0.149	0.682	0.136	0.121	0.713	0.28	3.89E-01
		BL vs. FU3	Left	0.097	0.099	0.643	0.070	0.058	0.641	0.04	4.82E-01
		BL vs. FU3	Right	0.129	0.099	0.697	0.083	0.098	0.667	-0.72	7.63E-01
		FU2 vs. FU3	Left	0.123	0.118	0.639	0.065	0.077	0.631	-0.12	5.49E-01
		FU2 vs. FU3	Right	0.097	0.111	0.732	0.082	0.072	0.654	0.33	3.70E-01
No Win	Discriminative	BL vs. FU2	Left	0.040	0.249	0.317	0.019	0.070	0.377	4.79	8.40E-07
		BL vs. FU2	Right	0.098	0.251	0.394	0.044	0.118	0.323	3.57	1.76E-04
		BL vs. FU3	Left	0.070	0.191	0.344	0.036	0.075	0.360	2.50	6.12E-03
		BL vs. FU3	Right	0.120	0.191	0.427	0.117	0.078	0.327	1.48	6.88E-02
		FU2 vs. FU3	Left	0.055	0.250	0.412	0.041	0.008	0.369	3.96	3.72E-05
		FU2 vs. FU3	Right	0.063	0.240	0.361	0.012	0.070	0.328	3.56	1.89E-04
	Shared	BL vs. FU2	Left	0.044	0.089	0.659	0.047	0.014	0.653	1.11	1.34E-01
		BL vs. FU2	Right	0.105	0.092	0.686	0.075	0.047	0.660	-0.34	6.32E-01
		BL vs. FU3	Left	0.067	0.113	0.677	0.077	0.073	0.657	1.05	1.48E-01
		BL vs. FU3	Right	0.129	0.088	0.705	0.053	0.091	0.589	-0.93	8.24E-01
		FU2 vs. FU3	Left	0.059	0.097	0.670	0.040	0.025	0.637	0.82	2.07E-01
		FU2 vs. FU3	Right	0.067	0.099	0.710	0.010	0.054	0.616	0.70	2.42E-01

Supplementary Table 6. Model-free principal component analysis (PCA) of bilateral M1 activations validates the dual-regulatory latent signals. Summary of the PCA extracting orthogonal signal components from the four mean activation values in the M1 hand regions (left/right hemisphere activations during left/right-hand responses). Data are presented across all longitudinal timepoints (Baseline [BL], Follow-up 2 [FU2], Follow-up 3 [FU3]) and reward conditions (Big Win [BW], Small Win [SW], No Win [NW]). The table details the specific region loadings, the explained variance ratio for each principal component (PC), and the Pearson correlation coefficients (r) and corresponding p-values between the model-free PCs and the MLR-derived shared and discriminative latent signals. The first component (PC_shared) exhibits approximately equal positive loadings across all four M1 regions, explains roughly half of the total variance (~44–48%), and is almost perfectly correlated with the model-based shared signal (r). The second component (PC_discriminative) captures the contralateral versus ipsilateral contrast, explains ~16–22% of the variance, and exhibits near-perfect correlation with the model-based discriminative signal. The remaining minor components (PC_hand, PC_hemi) account for residual inter-hand and inter-hemispheric variance. These model-free results independently corroborate the existence and orthogonality of the proposed shared and discriminative regulatory processes without relying on a priori model assumptions.

Timepoint	Condition	Component	Loading_L_Hand_L_M1	Loading_L_Hand_R_M1	Loading_R_Hand_L_M1	Loading_R_Hand_R_M1	Explained_Variance_Ratio	r_with_shared	p_with_shared	r_with_discriminative	p_with_discriminative
BL	BW	PC_shared	0.48	0.53	0.51	0.47	0.46	1.00	1.41E-2375	0.09	0.000184
		PC_discriminative	-0.49	0.45	0.51	-0.55	0.22	-0.01	0.623	0.99	1.12E-1417
		PC_hand	-0.71	-0.07	0.11	0.69	0.17	-0.01	0.766	0.06	0.0205
		PC_hemi	0.12	-0.72	0.68	-0.06	0.15	0.01	0.602	-0.02	0.467
	SW	PC_shared	0.49	0.51	0.51	0.50	0.46	1.00	1.28E-2241	-0.01	0.684
		PC_discriminative	-0.51	0.50	0.49	-0.50	0.20	-0.02	0.394	1	1.62E-2041
		PC_hand	-0.59	-0.40	0.39	0.59	0.18	0.00	0.925	0.02	0.508
		PC_hemi	0.39	-0.59	0.59	-0.39	0.16	0.03	0.191	0.04	0.116
	NW	PC_shared	0.49	0.51	0.49	0.51	0.48	1.00	5.75E-1978	-0.04	0.167
		PC_discriminative	-0.19	0.44	0.49	-0.73	0.16	0.00	0.953	0.9	9.29E-532
		PC_hand	-0.64	-0.32	0.59	0.36	0.19	0.00	0.874	0.32	9.23E-38
		PC_hemi	0.56	-0.66	0.41	-0.27	0.16	0.04	0.123	-0.23	5.64E-20
FU2	BW	PC_shared	0.53	0.47	0.50	0.49	0.45	1.00	1.5E-1623	-0.03	0.312
		PC_discriminative	-0.33	0.62	0.36	-0.61	0.20	0.02	0.427	0.95	1.1E-538
		PC_hand	-0.75	-0.12	0.39	0.52	0.16	-0.02	0.511	0.29	1.34E-22
		PC_hemi	0.22	-0.61	0.68	-0.34	0.18	0.03	0.356	0.1	0.000785
	SW	PC_shared	0.53	0.48	0.52	0.48	0.45	1.00	8.22E-1644	-0.01	0.698
		PC_discriminative	-0.15	0.52	0.36	-0.76	0.19	-0.01	0.676	0.88	2.24E-348
		PC_hand	-0.69	-0.20	0.63	0.29	0.16	0.04	0.199	0.44	3.50E-53
		PC_hemi	0.47	-0.67	0.46	-0.34	0.19	0.02	0.468	-0.14	4.98E-06
	NW	PC_shared	0.52	0.50	0.50	0.47	0.46	1.00	6.06E-1409	-0.04	0.246
		PC_discriminative	-0.68	0.47	0.49	-0.26	0.16	-0.02	0.438	0.97	1.86E-632
		PC_hand	-0.28	-0.67	0.50	0.48	0.19	-0.02	0.625	-0.17	1.05E-07
		PC_hemi	0.43	-0.29	0.50	-0.69	0.19	0.01	0.867	0.15	1.60E-06
FU3	BW	PC_shared	0.51	0.51	0.50	0.48	0.44	1.00	5.38E-1401	0.02	0.464
		PC_discriminative	-0.11	0.22	0.62	-0.75	0.20	0.02	0.582	0.83	1.21E-235
		PC_hand	-0.40	-0.57	0.61	0.39	0.18	0.00	0.940	0.07	0.0431
		PC_hemi	0.76	-0.61	0.08	-0.23	0.18	0.03	0.308	-0.49	1.56E-54
	SW	PC_shared	0.50	0.50	0.50	0.49	0.44	1.00	1.82E-1364	0.02	0.457
		PC_discriminative	-0.54	0.55	0.44	-0.45	0.19	0.01	0.866	0.99	3.85E-765
		PC_hand	-0.50	-0.50	0.52	0.49	0.17	0.02	0.599	0.01	0.775
		PC_hemi	0.46	-0.44	0.54	-0.56	0.20	0.03	0.344	0.14	2.26E-05
	NW	PC_shared	0.52	0.48	0.49	0.51	0.45	1.00	2.24E-1165	-0.09	0.0126
		PC_discriminative	-0.52	0.49	0.54	-0.44	0.17	0.01	0.880	0.99	1.15E-796
		PC_hand	-0.65	-0.20	0.14	0.72	0.18	-0.04	0.201	-0.02	0.467
		PC_hemi	0.18	-0.70	0.68	-0.16	0.20	-0.04	0.260	-0.06	0.083

Supplementary Table 7. Multivariate linear regression (MLR) of the lateralized "executive preparation" component (PC_discriminative) on mean RT of success trials. Each row represents an independent MLR model where the mean reaction time of successful trials (Y, standardized) was regressed simultaneously against the effector-specific PC_discriminative signals across the five motor regions (X, standardized), controlling for covariates (sites and sex). Standardized β coefficients and P values are reported. Highlighting a strictly localized functional architecture, the PC_discriminative signal of the M1 hand area profoundly dominates the prediction of execution speed, effectively neutralizing the predictive variance of broader motor/premotor regions when evaluated concurrently.

Timepoint	Condition	Hand	M1_Hand		preSMA		SMA		PMv		PMd		Model	
			β	*P*	β	*P*	β	*P*	β	*P*	β	*P*	F	*P*
BL	Big Win	mean RT left	-0.21	4.47E-13	-0.04	1.76E-01	0.01	6.51E-01	0.04	1.46E-01	0.03	3.65E-01	12.91	5.68E-29
		mean RT right	-0.23	1.02E-14	-0.01	6.93E-01	-0.01	6.33E-01	0.05	6.40E-02	0.03	3.26E-01	14.71	1.73E-33
	Small Win	mean RT left	-0.21	4.06E-14	-0.01	5.77E-01	0.00	9.36E-01	-0.01	6.67E-01	-0.01	7.26E-01	13.26	7.37E-30
		mean RT right	-0.25	4.11E-19	-0.02	5.56E-01	-0.03	3.47E-01	0.02	5.49E-01	0.00	8.87E-01	15.01	3.02E-34
	No Win	mean RT left	-0.19	6.36E-12	0.00	9.82E-01	0.03	2.55E-01	-0.02	5.81E-01	0.00	9.62E-01	12.36	1.63E-27
		mean RT right	-0.20	1.16E-12	-0.02	5.44E-01	0.04	1.85E-01	-0.02	5.62E-01	0.03	3.81E-01	9.80	4.31E-21
FU2	Big Win	mean RT left	-0.19	6.85E-10	-0.01	7.88E-01	0.00	9.72E-01	0.03	3.58E-01	-0.02	4.56E-01	25.38	2.34E-56
		mean RT right	-0.14	8.10E-06	0.00	9.92E-01	-0.01	8.30E-01	0.01	7.85E-01	-0.01	7.67E-01	23.07	2.45E-51
	Small Win	mean RT left	-0.25	2.32E-16	0.00	9.76E-01	-0.04	1.35E-01	0.02	5.85E-01	-0.02	5.21E-01	29.13	3.20E-64
		mean RT right	-0.23	4.69E-14	0.04	2.15E-01	-0.03	2.67E-01	-0.05	7.19E-02	0.02	4.57E-01	23.86	4.23E-53
	No Win	mean RT left	-0.18	2.66E-08	-0.03	3.84E-01	0.03	4.02E-01	-0.03	2.79E-01	-0.01	7.90E-01	19.36	8.82E-43
		mean RT right	-0.15	1.48E-06	0.02	5.45E-01	0.07	3.78E-02	-0.02	4.56E-01	0.01	7.03E-01	19.53	3.60E-43
FU3	Big Win	mean RT left	-0.17	2.61E-05	0.09	1.96E-02	0.03	4.45E-01	0.07	6.84E-02	-0.01	7.67E-01	11.06	1.72E-21
		mean RT right	-0.17	1.14E-05	0.08	3.42E-02	0.04	3.13E-01	0.05	1.66E-01	-0.04	3.33E-01	14.52	1.20E-28
	Small Win	mean RT left	-0.18	5.33E-06	-0.02	6.87E-01	0.03	4.25E-01	0.06	1.00E-01	-0.04	3.65E-01	11.33	4.40E-22
		mean RT right	-0.19	4.89E-07	-0.04	2.88E-01	0.07	6.57E-02	0.07	5.83E-02	-0.05	1.98E-01	16.01	1.01E-31
	No Win	mean RT left	-0.13	1.50E-03	0.05	2.37E-01	-0.01	7.76E-01	-0.04	2.93E-01	0.07	7.18E-02	8.18	3.13E-15
		mean RT right	-0.15	1.23E-04	0.07	7.14E-02	0.04	3.35E-01	-0.01	8.21E-01	0.02	5.52E-01	9.17	2.30E-17

Supplementary Table 8. Multivariate linear regression (MLR) of the shared "cognitive readiness" component (PC_shared) on global temporal precision. Each row represents an independent MLR model where a specific behavioral metric (Y, standardized) was regressed simultaneously against the PC_shared signals extracted from five key motor regions (M1hand, preSMA, SMA, PMv, and PMd; X, standardized). All models were adjusted for baseline covariates including sex and site. Values denote standardized beta coefficients and their corresponding P values. Aligning with its broad network engagement, the PC_shared component in M1 hand area demonstrate associations with global response precision when evaluated concurrently with other motor/premotor regions.

Timepoint	Condition	Metric	M1_Hand		preSMA		SMA		PMv		PMd		Model	
			β	*P*	β	*P*	β	*P*	β	*P*	β	*P*	F	*P*
BL	Big Win	mean RT	0.17	4.41E-07	0.25	2.63E-08	0.01	7.86E-01	0.03	3.46E-01	-0.27	1.01E-08	7.58	1.42E-15
		RT SD	-0.14	3.34E-05	-0.27	9.85E-10	-0.02	6.26E-01	-0.02	4.77E-01	0.27	1.33E-08	8.19	3.99E-17
		success rate	0.13	8.05E-05	0.04	3.71E-01	0.00	9.21E-01	0.03	3.26E-01	-0.14	2.47E-03	3.46	1.40E-05
		too-early rate	-0.17	6.24E-07	-0.27	5.85E-10	-0.03	5.63E-01	-0.05	1.03E-01	0.32	1.30E-11	9.17	1.38E-19
		too-late rate	0.02	4.60E-01	0.18	5.05E-05	0.06	1.97E-01	0.01	8.17E-01	-0.14	2.50E-03	4.33	1.40E-07
	Small Win	mean RT	0.13	6.96E-05	0.21	6.28E-07	-0.05	2.31E-01	0.04	2.20E-01	-0.20	1.75E-05	5.19	1.16E-09
		RT SD	-0.11	1.01E-03	-0.19	4.42E-06	0.00	9.31E-01	-0.03	3.59E-01	0.19	3.55E-05	2.05	1.22E-02
		success rate	0.06	8.59E-02	0.02	6.37E-01	0.01	7.98E-01	0.02	5.11E-01	-0.03	4.50E-01	5.59	1.23E-10
		too-early rate	-0.10	2.89E-03	-0.24	1.21E-08	0.03	5.11E-01	-0.04	2.74E-01	0.21	3.46E-06	4.83	8.78E-09
		too-late rate	0.07	4.52E-02	0.16	1.29E-04	0.01	7.53E-01	-0.01	7.40E-01	-0.16	4.63E-04	5.53	1.76E-10
	No Win	mean RT	0.15	1.00E-05	0.17	1.53E-04	0.00	9.58E-01	0.04	2.94E-01	-0.17	1.80E-04	6.32	2.15E-12
		RT SD	-0.13	6.62E-05	-0.19	2.17E-05	-0.03	4.78E-01	-0.01	8.22E-01	0.20	1.15E-05	7.23	1.13E-14
		success rate	-0.02	5.94E-01	-0.09	4.07E-02	0.09	4.48E-02	0.01	8.18E-01	-0.05	2.54E-01	1.95	1.87E-02
		too-early rate	-0.10	4.62E-03	-0.19	2.24E-05	-0.03	5.07E-01	-0.05	1.32E-01	0.23	9.24E-07	5.20	1.17E-09
		too-late rate	0.14	6.69E-05	0.11	1.18E-02	0.03	4.55E-01	0.04	2.78E-01	-0.13	5.49E-03	5.40	3.89E-10
FU2	Big Win	mean RT	0.14	3.64E-04	0.24	5.71E-07	-0.04	4.09E-01	0.04	2.91E-01	-0.25	1.60E-06	7.85	6.18E-16
		RT SD	-0.15	1.70E-04	-0.26	2.67E-08	0.02	7.06E-01	-0.07	8.72E-02	0.27	8.15E-08	8.43	2.26E-17
		success rate	0.12	4.47E-03	0.10	4.04E-02	0.05	2.67E-01	-0.02	5.90E-01	-0.13	1.03E-02	4.08	6.22E-07
		too-early rate	-0.17	1.10E-05	-0.27	8.15E-09	0.00	9.94E-01	-0.02	5.69E-01	0.26	2.16E-07	7.55	3.23E-15
		too-late rate	0.04	3.46E-01	0.12	1.32E-02	-0.01	8.35E-01	0.07	8.89E-02	-0.11	4.19E-02	2.75	5.19E-04
	Small Win	mean RT	0.02	5.87E-01	0.15	2.24E-03	0.04	4.83E-01	0.09	2.72E-02	-0.19	5.70E-04	2.91	2.46E-04
		RT SD	-0.01	8.72E-01	-0.10	4.15E-02	-0.05	3.74E-01	-0.12	3.19E-03	0.17	1.18E-03	3.53	1.12E-05
		success rate	-0.02	5.59E-01	-0.11	3.38E-02	0.10	5.62E-02	0.02	6.64E-01	-0.02	6.85E-01	1.21	2.61E-01
		too-early rate	-0.11	6.47E-03	-0.18	3.64E-04	-0.03	5.46E-01	-0.08	6.23E-02	0.22	3.27E-05	4.58	4.27E-08
		too-late rate	0.12	3.04E-03	0.17	5.93E-04	-0.04	4.20E-01	0.07	7.89E-02	-0.13	1.29E-02	3.24	4.68E-05
	No Win	mean RT	0.07	9.04E-02	0.11	3.73E-02	-0.06	2.97E-01	0.06	1.56E-01	-0.14	1.32E-02	2.63	9.52E-04
		RT SD	-0.05	2.38E-01	-0.14	1.05E-02	0.01	8.82E-01	-0.03	4.68E-01	0.18	1.51E-03	2.91	2.44E-04
		success rate	-0.05	1.84E-01	-0.07	2.03E-01	0.08	1.23E-01	-0.07	1.20E-01	0.11	4.94E-02	1.76	4.07E-02
		too-early rate	-0.08	4.60E-02	-0.18	1.07E-03	0.04	4.95E-01	-0.06	2.03E-01	0.19	6.25E-04	2.60	1.06E-03
		too-late rate	0.11	5.44E-03	0.07	1.74E-01	0.06	2.67E-01	0.08	5.13E-02	-0.22	6.12E-05	4.18	3.78E-07
FU3	Big Win	mean RT	0.12	1.77E-02	0.14	2.00E-02	0.04	5.39E-01	0.05	3.33E-01	-0.16	1.10E-02	4.52	2.15E-07
		RT SD	-0.14	4.12E-03	-0.17	3.42E-03	-0.08	2.02E-01	0.04	4.41E-01	0.13	4.50E-02	4.83	4.59E-08
		success rate	0.06	2.36E-01	0.05	3.93E-01	0.00	9.79E-01	0.01	8.07E-01	-0.02	8.09E-01	2.07	1.43E-02
		too-early rate	-0.14	3.17E-03	-0.15	8.69E-03	-0.05	3.97E-01	-0.10	7.04E-02	0.17	6.50E-03	4.93	2.85E-08
		too-late rate	0.10	3.13E-02	0.11	6.05E-02	0.00	9.91E-01	0.02	7.58E-01	-0.13	4.29E-02	3.93	3.54E-06
	Small Win	mean RT	0.01	7.93E-01	0.01	9.09E-01	0.12	5.46E-02	0.02	7.60E-01	-0.08	2.12E-01	1.89	2.83E-02
		RT SD	-0.02	7.00E-01	-0.01	8.26E-01	-0.10	1.03E-01	-0.01	8.09E-01	0.08	2.09E-01	1.29	2.12E-01
		success rate	0.01	8.99E-01	-0.01	8.69E-01	0.01	8.60E-01	-0.05	3.87E-01	0.10	1.19E-01	1.24	2.48E-01
		too-early rate	-0.01	8.09E-01	-0.09	1.73E-01	-0.19	3.47E-03	-0.01	7.95E-01	0.14	2.72E-02	2.76	7.98E-04
		too-late rate	0.02	7.22E-01	0.00	9.57E-01	0.18	4.03E-03	0.05	3.03E-01	-0.22	5.40E-04	2.50	2.38E-03
	No Win	mean RT	0.13	1.01E-02	0.18	4.52E-03	0.01	8.54E-01	-0.08	1.48E-01	-0.11	7.85E-02	3.43	3.84E-05
		RT SD	-0.15	1.63E-03	-0.21	6.71E-04	0.02	7.57E-01	0.08	1.16E-01	0.15	1.90E-02	4.23	8.96E-07
		success rate	0.05	3.22E-01	-0.06	3.65E-01	-0.07	2.81E-01	-0.03	5.40E-01	0.12	5.44E-02	2.22	7.83E-03
		too-early rate	-0.14	4.16E-03	-0.29	3.35E-06	0.03	5.92E-01	0.03	5.44E-01	0.16	8.76E-03	4.78	6.26E-08
		too-late rate	0.09	5.79E-02	0.19	2.09E-03	0.03	6.89E-01	0.05	3.68E-01	-0.24	1.92E-04	2.76	7.97E-04

Supplementary Table 9. Context-dependent modulations of task performance by temporal variability. Comparison of response outcomes (Success, Too-Early, and Too-Late rates) across distinct temporal anticipation contexts in the ZIB cohort. Trials were categorized by the difference in delay duration from the preceding trial: Compressed, Consistent, and Extended. Values denote Mean +/- SD. P-values were derived from two-sided paired t-tests comparing the perturbed contexts against the Consistent trials.

Metric	Comparison	Compressed (Mean±SD)	Consistent (Mean±SD)	Extended (Mean±SD)	t-value	P-value
Success Rate	Compressed vs Consistent	48.9% ± 10.4%	66.8% ± 9.8%	-	-57.55	2.57E-462
	Consistent vs Extended	-	66.8% ± 9.8%	60.2% ± 16.5%	16.18	4.11E-56
	Compressed vs Extended	48.9% ± 10.4%	-	60.2% ± 16.5%	-27.26	1.11E-143
Too-Early Rate	Compressed vs Consistent	2.1% ± 3.3%	5.9% ± 5.3%	-	-31.55	1.43E-184
	Consistent vs Extended	-	5.9% ± 5.3%	12.5% ± 12.6%	-25.27	7.49E-126
	Compressed vs Extended	2.1% ± 3.3%	-	12.5% ± 12.6%	-40.25	2.72E-274
Too-Late Rate	Compressed vs Consistent	49.0% ± 10.7%	27.3% ± 9.7%	-	69.81	1.67E-592
	Consistent vs Extended	-	27.3% ± 9.7%	27.3% ± 16.2%	0.12	0.906
	Compressed vs Extended	49.0% ± 10.7%	-	27.3% ± 16.2%	53.42	2.3E-417
